# Benefits of high-dose intravenous immunoglobulin on mortality in patients with severe COVID-19: An updated systematic review and meta-analysis

**DOI:** 10.3389/fimmu.2023.1116738

**Published:** 2023-01-23

**Authors:** Xiaosheng Liu, Yuelun Zhang, Lianfeng Lu, Xiaodi Li, Yuanni Wu, Yang Yang, Taisheng Li, Wei Cao

**Affiliations:** ^1^ Tsinghua-Peking Center for Life Sciences, Beijing, China; ^2^ Department of Basic Medical Sciences, School of Medicine, Tsinghua University, Beijing, China; ^3^ Department of Infectious Diseases, Peking Union Medical College Hospital, Peking Union Medical College and Chinese Academy of Medical Sciences, Beijing, China; ^4^ Medical research center, Peking Union Medical College Hospital, Peking Union Medical College and Chinese Academy of Medical Sciences, Beijing, China; ^5^ State Key Laboratory of Complex Severe and Rare Diseases, Peking Union Medical College Hospital, Chinese Academy of Medical Science and Peking Union Medical College, Beijing, China

**Keywords:** COVID-19, IVIg, systemic review, meta-analysis, meta-regression

## Abstract

**Background:**

The clinical benefits of high-dose intravenous immunoglobulin (IVIg) in treating COVID-19 remained controversial.

**Methods:**

We systematically searched databases up to February 17, 2022, for studies examining the efficacy of IVIg compared to routine care. Meta-analyses were conducted using the random-effects model. Subgroup analysis, meta-regression, and trial series analysis w ere performed to explore heterogeneity and statistical significance.

**Results:**

A total of 4,711 hospitalized COVID-19 patients (1,925 IVIg treated and 2786 control) were collected from 17 studies, including five randomized controlled trials (RCTs) and 12 cohort studies. The application of IVIg was not associated with all-cause mortality (RR= 0.89 [0.63, 1.26], P= 0.53; I^2^ = 75%), the length of hospital stays (MD= 0.29 [-3.40, 6.44] days, P= 0.88; I2 = 96%), the needs for mechanical ventilation (RR= 0.93 ([0.73, 1.19], P= 0.31; I2 = 56%), or the incidence of adverse events (RR= 1.15 [0.99, 1.33], P= 0.06; I2 = 20%). Subgroup analyses showed that overall mortality among patients with severe COVID-19 was reduced in the high-dose IVIg subgroup (RR= 0.33 [0.13, 0.86], P= 0.02, I^2^ = 68%; very low certainty).

**Conclusions:**

Results of this study suggest that severe hospitalized COVID-19 patients treated with high-dose IVIg would have a lower risk of death than patients with routine care.

**Systematic review registration:**

https://www.crd.york.ac.uk/prospero/display_record.php?ID=CRD42021231040, identifier CRD42021231040.

## 1 Introduction

The global pandemic of coronavirus disease 2019 (COVID-19) caused by the severe acute respiratory syndrome coronavirus 2 (SARS-CoV-2) infection has become the major public health concern over the past three years. As of 1 November 2022, 627 million confirmed cases and 6.5 million deaths have been reported globally (1). Although the majority of COIVD-19 patients present a mild, moderate, or even asymptomatic disease course, 10-20% of patients would develop into severe and critically ill status with a higher risk of mortality (2). The hyperinflammatory state is an outstanding hallmark during the disease course of severe and critically ill COVID-19 patients. The dysregulated innate immune responses are considered to contribute to immunopathological inflammation, inspiring the application of immunomodulators in treating COVID-19 patients (2–6).

Intravenous immunoglobulin (IVIg) preparation is a purified blood product with multifunctional immunomodulatory properties (7–9). We firstly reported the clinical effectiveness of high-dose IVIg (0.3-0.5 g/kg/day for five days) on three deteriorating COVID-19 patients in China (10). The clinical application of IVIg was subsequently reported by another Iranian group in treating five COVID-19 patients (11). Moreover, the administration of high-dose of IVIg did help to reduce the plasmatic levels of several cytokines and chemokines (12). In addition to managing COVID-19 as a whole, IVIg therapy was also applied in treating neurologic complications associated with COVID-19, such as COVID-19-related encephalopathy (13), COVID-19-trigged Guillain-Barré Syndrome (GBS) (14), and acute demyelinating encephalomyelitis associated with COVID-19 (15). IVIg therapy was also involved in treating vaccine-induced immune thrombotic thrombocytopenia (VITT) (16) and post-acute sequelae of SARS-CoV-2 infection (PASC) (17).

However, the following results from different randomized controlled trials (RCTs) and cohort studies were inconsistent. A double-blind RCT demonstrated that administration of IVIg (20 g/day for three days) could significantly reduce the mortality rate of severe COVID-19 patients (18), while another RCT study (IVIg dosage: 0.4 g/kg/day for three days) didn’t observe any difference between IVIg groups and control groups in mortality or the need for mechanical ventilation (19). Two retrospective NRSI studies observed that IVIg could significantly reduce mortality rate and decrease the inflammatory response in critically ill COVID-19 patients (20, 21), while the recent RCT study showed that IVIg did not improve the clinical outcomes of COVID-19 patients with moderate-to-severe acute respiratory distress syndrome (ARDS) (22). The clinical consensus on applying IVIg could not be unified due to the inconsistency of different studies. The controversy over the definite clinical efficacy of IVIg on severe COVID-19 patients remained in debate (23, 24).

Evaluating the efficacy and safety of IVIg in COVID-19 patients is important to assist physicians’ clinical decision-making and promote a favorable prognosis for patients. Meta-analysis is a quantitative statistical method widely used to synthesize available clinical evidence included within systematic review (25, 26). Three published meta-analyses focused on the clinical efficacy of IVIg in treating COVID-19 (27–29). These studies provided valuable information, although they applied crude death rates, which had been discouraged in meta-analysis (30, 31). Here, we aimed to provide an up‐to‐date systematic review and meta-analysis of the efficacy and safety of IVIg in the treatment of hospitalized COVID-19 patients compared to standard care. We investigated whether high-dose IVIg would reduce mortality in hospitalized severe COVID-19 patients, and further conducted meta-regression and trial-series analysis to explore the potential heterogeneity.

## 2 Methods

The systematic review and meta-analysis followed the request from the Preferred Reporting Items for Systematic Review and Meta-Analyses (PRISMA) guidelines (32), the protocol of this systematic review was pre-registered in the International Prospective Register of Systematic Reviews (PROSPERO) database before literature search (ID: CRD42021231040). The detailed protocol changes were listed in **Table S1**.

### 2.1 Search strategy and selection criteria

According to the previous protocol, we performed a systematic literature search for clinical trials and observational studies on the following databases and registers: Embase, PubMed, ClinicalTrials.gov, WHO COVID-19 Global literature on coronavirus disease, Cochrane Central Register of Controlled Trials, Scopus, Web of Science, medRxiv, bioRxiv and ArXiv. The searching strategy used subject and free-text terms covering (“intravenous immunoglobulin” or “immunoglobulin” or “intravenous Ig” or “IVIg” or “IgIV”) and (“COVID-19” or “2019-nCoV disease” or “Coronavirus Disease 2019” or “SARS-CoV-2”) in each database. The publish date of studies was restricted between December 30, 2019 and February 17, 2022, with no language limitation.

The inclusion criteria included (1): Randomized controlled clinical trials and cohort-controlled trials that reported the efficacy of IVIg; (2) Hospitalized patients with lab-confirmed SARS-CoV-2 infection. The exclusion criteria included: (1) Review articles, editorials, opinions, case report, case series; (2) Single-arm study with IVIg intervention only; (3) Studies that examined the efficacy of IVIg on pediatric patients; (4) Studies which applied the non-standard IVIg preparation (e.g., hyperimmune IVIg or IgM-enriched IVIg, et al.). Two authors (LF.L and XD.L) conducted the literature screening independently, titles, abstracts of citations, and full-text, if appropriate, were accessed to determine whether they met the eligibility criteria above. Citations with duplicates or disagreements were solved by another author (XS.L). Further uncertainties about inclusion were resolved by two senior authors (W.C and TS.L).

### 2.2 Outcomes of interest

The primary outcome of interest of this study was the overall mortality among hospitalized COVID-19 patients. The secondary outcome of interest included the length of hospital stay, the needs for mechanical ventilation, the incidence of adverse events (AEs) and serious adverse events (SAEs).

### 2.3 Data extraction and risk of bias assessment

Data on first author name, publish date, type of study, the severity of disease, number of participants, countries of study of each study were extracted. According to the Cochrane Handbook, the Revised Cochrane tool for assessing the risk of bias in randomized trials (RoB 2.0) was applied to evaluate the risk of bias among included RCT studies (33). The Risk of Bias in Non-randomized Studies-of Interventions tool (ROBINS-I) and the Newcastle-Ottawa Scale (NOS) were applied to evaluate the risk of bias among included cohort studies (34, 35). The NOS scale includes 8 items in 3 domains, with a total of 9 scores. Studies with scores ≥ 6 are considered to be of high quality and low risk. The level of evidence was judged according to the Cochrane’s Grading of Recommendations, Assessment, Development and Evaluation (GRADE) assessment handbook (36).

### 2.4 Statistical analysis

Meta-analysis was conducted on at least two studies with combinable outcome indicators. Dichotomous variables (including all-cause mortality, the need for mechanical ventilation, the incidences of AE and SAE) were calculated using relative risk (RR) with 95% confidence interval (CI), and continuous variables (e.g., the length of hospital stay) were calculated using mean difference (MD) with 95% CI. Continuous variables reported with median and interquartile range (IQR) were converted into mean and standard deviation (SD) using the online tool (https://www.math.hkbu.edu.hk/~tongt/papers/median2mean.html) (37–39). If the unadjusted and adjusted RRs were reported in cohort studies, the adjusted effects were used for data pooling. The random-effects model was used to calculate effect sizes and the summarized results were shown as forest plots, respectively. The Q test and I^2^ index were used to calculate the heterogeneity of the integrated results (Not significant heterogeneity: I^2^< 40%; moderate heterogeneity: 40%-70%; substantial heterogeneity: I^2^> 70%). For evaluating the risk of publication bias, the inverted funnel-plot analysis and Egger’s test were conducted.

Subgroup analyses and meta-regression were used to explore the source of heterogeneity. Subgroup analyses based on study design and disease severity (critically ill/severe/non-severe) were conducted. The definitions of disease severity followed the WHO clinical management guideline (40). Critically ill COVID-19 was defined by the presence for ARDS, sepsis, septic shock, or need for mechanical ventilation (40). Severe COVID-19 was defined by any of the following indicators not in conformity with critically-ill: respiratory distress (respiratory rate ≥ 30 breaths/min), oxygen saturation (SpO_2_) ≤ 90% at rest on room air, or signs of pneumonia and severe respiratory distress (40). Non-severe COVID-19 was defined as the absence of any symptoms for critically ill and severe type (40).

The maximum likelihood random-effects meta-regression was conducted to explore the association of mortality with sample size, age, sex, the incidence of hypertension or diabetes, the percentage of corticosteroids usage, the daily dosage of IVIg (high-dose [defined as 0.4-1.0 g/kg/day] or low-dose [less than 0.4 g/kg/day], except for one study that applied 15 g/day as the threshold (20), and the duration of IVIg therapy of the participants from each study. Regression coefficient (Coef.) was calculated to represent the correlation. Variables with *P*-value < 0.1 in univariable analysis were selected into further multivariable analysis. The trial series analysis (TSA) was performed to determine the two-sided conventional test boundaries, O’Brien-Fleming statistical significance boundaries, futility boundaries. The control event proportion was obtained from included studies. All *P*-values in this study were two-tailed, and statistical significance was set at *P* < 0.05. All analyses were performed using Review Manager (RevMan, version 5.3, the Cochrane Collaboration, UK), STATA (version 16.0, StataCorp LLC, USA) and the TSA software (version 0.9.5.10 Beta; Copenhagen Trial Unit, Copenhagen, Denmark).

## 3 Results

### 3.1 Study characteristics

**Figure 1:**
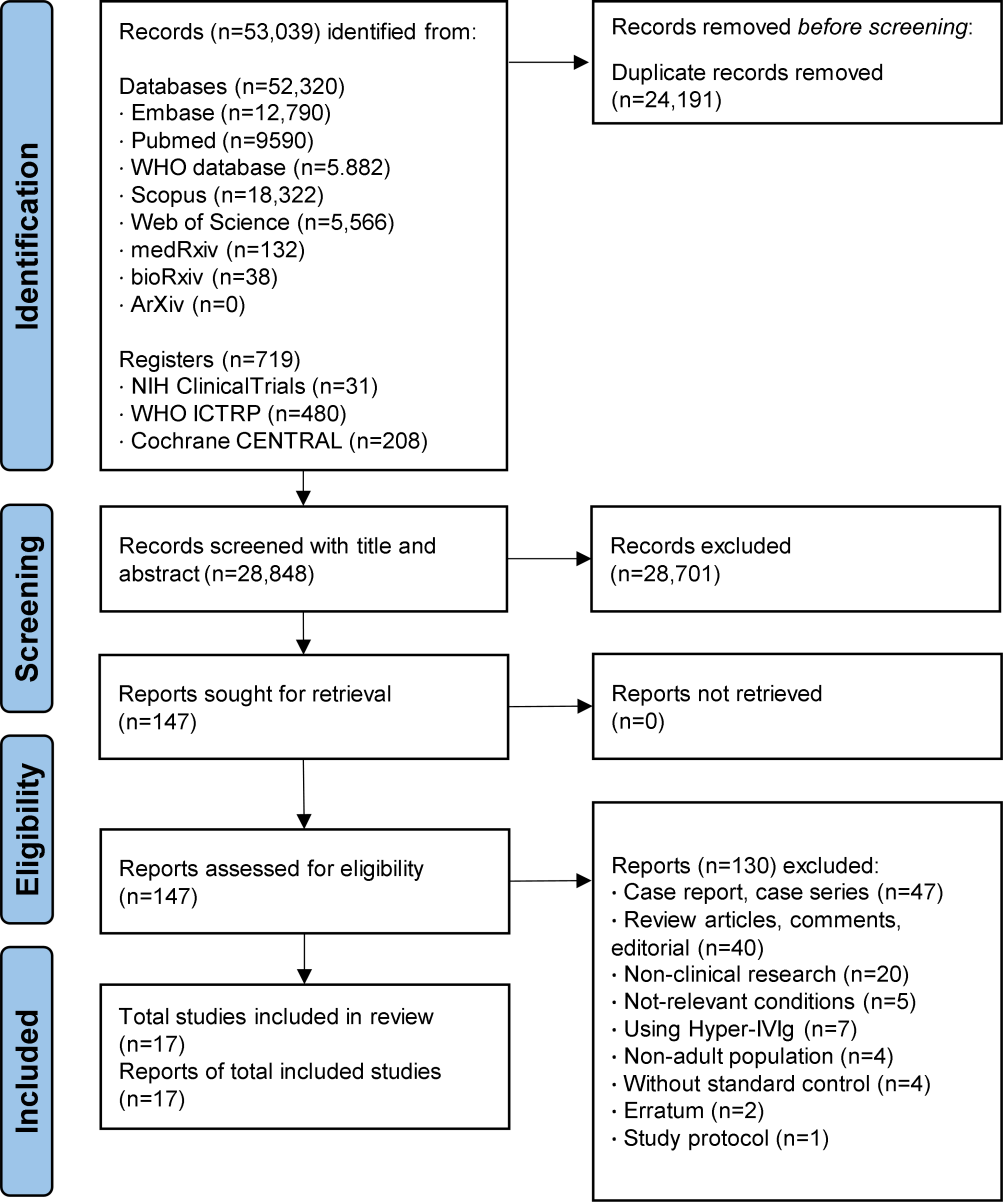
Prisma flow chart of included studies.

As shown in **Figure 1**, a total of 53,039 records from electronic literature databases were identified through the initial searching process. At the first eligibility check stage on title and abstract, 147 articles were considered as potentially eligible and were assessed for full-text for further screening. After screening, 17 studies (including five RCT (18, 19, 22, 41, 42) and 12 cohort studies (20, 21, 43–52) that were considered to meet the selection criteria and included in the meta-analysis. Different form the latest study (28), four studies were newly identified and included in this meta-analysis (48–50, 52).

A total of 4,711 hospitalized COVID-19 patients were included in this study, and 1,925 (40.9%) of them were treated by IVIg (**Table 1**). Among the 17 studies, six studies were conducted in China, four were conducted in Iran, two were conducted in India or Turkey, respectively. The dosage and duration of IVIg were greatly variable between studies. Of note, the average age of participants ranged from 37 to 68 years old (**Table S2**). Also, the percentage of male ranged from 33.0% to 94.9%, the incidence of hypertension or diabetes ranged from 20.2% to 58.5% and 11.5% to 53.0% across different studies, showing the internal discrepancies in demographic characteristics and comorbidities.

**Table 1:** Characteristics of included studies for meta-analysis.

First author, Year [Ref.]	Study Design	Disease Severit*y*	Country	Sample size (IVIg/control)	IVIg Daily Dosage	IVIg Duration	Control intervention
Gharebaghi, 2020 (18)	RCT	Severe	Iran	30/29	20 g/d	3 days	Routine care + placebo
Sakoulas, 2020 (41)	RCT	Severe	U.S.A.	16/17	0.5g/kg/d	3 days	Routine care
Tabarsi, 2021 (19)	RCT	Severe	Iran	52/32	0.4 g/kg/d	3 days	Standard care^a^
Raman, 2021 (42)	RCT	Non-severe	India	50/50	0.4 g/kg/d	5 days	Standard care^b^
Mazeraud, 2022 (22)	RCT	Critically ill	France	69/77	0·5 g/kg/d	4 days	Routine care+ placebo
Shao, 2020 (20)	Cohort	Severe and critically ill^c^	China	174/151	0.1-0.5 g/kg/d^d^	5-15 days^d^	Routine care
Esen, 2021 (21)	Cohort	Critically ill	Turkey	51/42	30 g/d	5 days	Standard care^e^
Farrokhpour, 2021 (51)	Cohort	Severe	Iran	23/43	0.4 g/kg/d	3–5 days	Standard care^f^
Cao, 2021 (44)	Cohort	Severe	China	26/89	0.4-1g/kg/d^d^	2-5 days^d^	Routine care
Huang, 2021 (43)	Cohort	Non-severe	China	45/594	10 and 20 g/d^f^	3 and 5 days^g^	Routine care
Liu, 2021 (45)	Cohort	Severe	China	421/429	10 (10–10) g/d^g^	9.5 (4–12) days^h^	Routine care
Hou, 2021 (46)	Cohort	Severe	China	47/66	N.A.	N.A.	Routine care
Ali, 2021	Cohort	Critically ill	Qatar	190/400	0.4 g/kg/d	4 (3–5) days^h^	Routine care
Kilic, 2021 (52)	Cohort	Severe	Turkey	10/16	0.3-0.5 g/kg/d	5 days	Routine care
Chen, 2021 (49)	Cohort	Critically ill	China	392/362	0.5 g/kg/d	N.A.	Routine care
Salehi, 2022 (48)	Cohort	Critically ill	Iran	74/109	0.25, 0.5 and 1 g/kg/d^i^	3-5 days^d^	Standard care^j^
Aggarwal, 2022 (50)	Cohort	Severe and critically ill^k^	India	255/280	0.5 g/kg/d	3 days	Routine care

RCT, randomized controlled trial; N.A., not available.

a Standard care consisted of oxygen and fluid support; lopinavir/ritonavir (200/50 mg), two tablets twice a day; and hydroxychloroquine 200 mg two times daily.

b Standard care consisted of azithromycin; lopinavir/ritonavir; piperacillin + tazobactam; acetaminophen and antacid.

c Among 174 IVIg treated patients, 103 were severe type and 71 were critically ill type.

d Data were represented as a range.

e Standard care consisted of hydroxychloroquine (800 mg loading dose, LD; 400 mg/day maintenance dose, MD, for 5 days), favipiravir (3200 mg LD; 1200 mg/day MD for 5 days), azithromycin (500 mg LD; 250 mg/day MD for 5 days), oseltamivir (150 mg/day for 5 days), tocilizumab or anakinra depending on inflammatory markers, methylprednisolone (200 mg/day), high dose vasopressors in case of septic shock and vitamin C (6 g/day i.v. for 7 days).

f Standard care consisted of oeltamivir + hydroxychloroquine + lopinavir/ritonavir or sofosbuvir or atazanavir ± ribavirin).

g 8 patients were treated 10 g/day for 3 days, 13 for 10 g/day for 5 days, 16 for 20 g/day for 3 days, and 8 for 20 g/day for 5 days.

h Data were represented as median and the interquartile range (IQR).

i 25 patients were treated in 0.25 g/kg/day, 32 in 0.5 g/kg/day, and 17 in 1 g/kg/day.

j Standard care consisted of oral hydroxychloroquine (HCQ) 400 mg daily for 5 days plus atazanavir/ritonavir (300/100) daily for 10 days.

k Among 255 IVIg treated patients, 175 were severe type and 74 were critically ill type.

### 3.2 Clinical effects of IVIg

**Figure 2:**
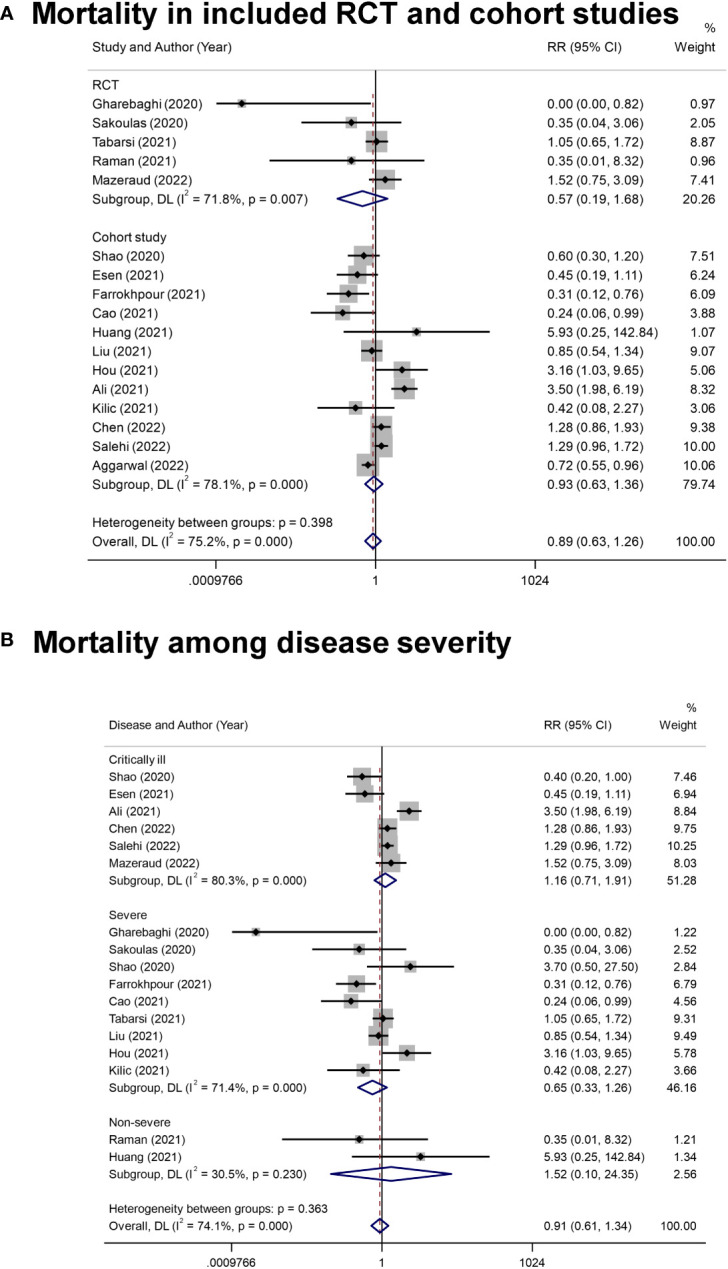
The forest plot of mortality. **(A)**, All-cause mortality in COVID-19 patients from included randomized controlled trial (RCT) and cohort studies. **(B)**, The mortality in COVID-19 patients with different disease severity.

The reported outcomes of included studies were summarized in **Table 2**. Overall, the application of IVIg did not reduce the mortality in RCT studies (RR= 0.57 [0.19, 1.68], *P*= 0.30; I^2^ = 72%), or cohort studies (RR= 0.93, [0.63, 1.36], *P*= 0.71; I^2^ = 78%) (**Figure 2A**). As there were five cohort studies that reported more than one adjusted effect estimated with different statistical methods (20, 44, 45, 47, 49), we additionally calculated the pooled RR of cohort studies as sensitive analysis (adjusted RR= 0.91, [0.67, 1.26], *P*= 0.58; I^2^ = 81%) (**Figure S1A**). These data suggested no significant improvement of IVIg on mortality of overall COVID-19 patients, and the certainty level was low due to the inconsistency in design bias and outcome measurement of studies. (**Table 2**).

**Table 2:** The GRADE assessment of reported outcomes.

Certainty assessment						Summary of findings			
No. of studies	Study design	Risk of bias	Inconsistency	Indirectness	Imprecision	No. of patients		Effect	Certainty
						IVIg	Control	Estimates (95%CI)	
Mortality- Overall patients									
17	5 RCT+ 12 Cohort	Serious	Serious	Not serious	Not serious	635/1850 (34.3%)	678/1999 (33.9%)	RR 0.89 (0.63 to 1.26)	⊕⊕◯◯ Low^a,c^
Critically ill patients									
6	1 RCT+ 5 Cohort	Serious	Serious	Not serious	Not serious	355/590 (60.2%)	336/585 (57.4%)	RR 1.16 (0.71 to 1.91)	⊕⊕◯◯ Low^a,c^
Severe patients									
9	3 RCT+ 6 Cohort	Serious	Serious	Not serious	Not serious	219/728 (30.1%)	256/840 (30.5%)	RR 0.65 (0.33 to 1.26)	⊕⊕◯◯ Low^a,c^
Severe patients with high-dose IVIg									
7	3 RCT+ 4 Cohort	Serious	Serious	Not serious	Serious	41/191 (21.5%)	94/345 (27.2%)	RR 0.33 (0.13 to 0.84)	⊕◯◯◯ Very low^b,d^
Non-severe patients									
2	1 RCT+ 1 Cohort	Serious	Not serious	Not serious	Serious	1/92 (1.1%)	1/139 (0.7%)	RR 1.52 (0.10 to 24.35)	⊕◯◯◯ Very low^b,e^
Length of hospital stay									
10	5 RCT+ 6 Cohort	Serious	Serious	Not serious	Not serious	539	656	MD 0.29 (-3.40 to 3.99)	⊕⊕◯◯ Low^a,c^
Severe patients									
8	2 RCT+ 6 Cohort	Serious	Serious	Not serious	Not serious	307	411	MD 2.62 (0.25 to 4.99)	⊕⊕◯◯ Low^a,c^
Need for mechanical ventilation									
8	3 RCT+ 5 Cohort	Serious	Serious	Not serious	Not serious	209/562 (37.2%)	244/669 (36.5%)	RR 0.95 (0.73 to 1.25)	⊕⊕◯◯ Low^a,c^
Adverse events- Overall patients									
6	3 RCT+ 3 Cohort	Serious	Serious	Not serious	Serious	173/323 (53.6%)	195/944 (20.7%)	RR 1.15 (0.99 to 1.33)	⊕◯◯◯ Very low^b,f^
Serious adverse events									
4	3 RCT+ 1 Cohort	Serious	Serious	Not serious	Not serious	22/160 (13.8%)	16/232 (6.9%)	RR 1.56 (0.89 to 2.73)	⊕◯◯◯ Very low^b,g^

No., number; RCT, randomized controlled trial; CI, confidence interval; RR, risk ratio; MD, mean difference.

a Low quality: the estimation of certainty was low, and further research might likely have an important impact on the estimate of effects.

b Very low quality: the estimation of certainty was uncertain.

c Downregulated 2 degrees for risk of bias and inconsistency.

d Downregulated 3 degrees for risk of bias, inconsistency, and imprecision (i.e., the precise IVIg daily dosage).

e Downregulated 3 degrees for risk of bias, the limited number of studies, and imprecision (i.e., the wide range of 95% CI).

f Downregulated 3 degrees for risk of bias, inconsistency, and imprecision (i.e., the precise incidence of AEs).

g Downregulated 3 degrees for risk of bias, inconsistency, and limited number of studies.

Since the clinical states caused great differences in prognosis, we conducted subgroup analysis on different disease severity. Of note, there were two studies examining the effects of IVIg in patients with severe and critically ill type (20, 50), and one was excluded in this analysis for not providing the results of subgroups (50). Results showed that IVIg did not reduce the mortality in different groups (Critically ill type: RR= 1.16 [0.71, 1.91], *P*= 0.56, I^2^ = 80%; Severe type: RR= 0.65 [0.33, 1.26], *P*= 0.20, I^2^ = 71%; Non-severe type: RR= 1.52 [0.10, 24.35], *P*= 0.77, I^2^ = 30%; *P* for interaction= 0.363) (**Figure 2B**). We also conducted subgroup analysis based on the differentially reported adjusted RR from four cohort studies (44, 45, 47, 49), with similar results (**Figure S1B**).

Effects of IVIg on the length of hospital stay were reported in 11 studies, including five RCT studies (18, 19, 22, 41, 42) (MD= -2.60 [-8.98, 3.77] days, *P*= 0.42; I^2^ = 97%) and six cohort studies (20, 43, 44, 46) (MD= 2.87 [-0.69, 6.44] days, *P*= 0.11; I^2^ = 88%) (**Figure S2A**). The integrated MD was 0.29 [-3.40, 6.44] days (*P*= 0.88; I^2^ = 96%; low quality of certainty) (**Table 2**). We found that the IVIg treatment might prolong the hospital stay of severe patients (MD = 2.62 [0.25, 4.99] days, *P*= 0.03; I^2^ = 79%), and low certainty level. (**Figure S2**; **Table 2**).

Effects of IVIg on the need for mechanical ventilation were reported in eight studies, including three RCT studies (19, 41, 42) (RR= 0.77 [0.49, 1.21], *P*= 0.26; I^2^ = 39%) and five cohort studies (21, 44, 46, 48, 50) (RR= 1.02 [0.76, 1.37], *P*= 0.89; I^2^ = 66%) (**Figure S3**). One RCT study was excluded in this analysis because all patients had applied mechanical ventilation before allocating to receive IVIg treatment (22). The pooled RR was 0.93 ([0.73, 1.19], *P*= 0.31; I^2^ = 56%), indicating no significant improvement of IVIg on the use of mechanical ventilation (**Figure S3**; **Table 2**).

### 3.3 Safety of IVIg

Safety of IVIg on the incidence of AE was reported in six studies, including three RCT studies (22, 41, 42) and three cohort studies (43, 44, 47). The overall incidence of AE and SAE were similar between groups [AE RR= 1.15 [0.99, 1.33], *P*= 0.06; I^2^ = 20%; low quality of certainty; SAE (RR= 1.56 [-0.89, 2.73], *P*= 0.12; I^2^ = 0%; low quality of certainty] (**Figure S4**; **Table 2**). Notable, the overall AE was might be underestimated since two studies only reported the incidence of acute kidney injury, instead of other AEs (43, 47). Meanwhile, the incidence of specific AE was unable to be further analyzed as not all studies reported the detailed list of AEs.

### 3.4 Meta-regression

Considering the internal discrepancies among studies, the meta-regression was further conducted to explore the source of heterogeneity. In the univariable meta-regression analyses, IVIg daily dosage (high or low) was considered as the risk factor in impacting mortality [Coef.= -0.34 (-0.57, 0.01), *P*= 0.004] (**Table 3**). The further multivariable meta-regression showed that IVIg daily dosage and duration accounted for 66.7% of heterogeneity and the daily dosage of IVIg remained statistically significant in multivariable meta-regression (**Table 3**).

**Table 3:** The results of univariable and multivariable meta regression on mortality rate.

Modulators	Univariable meta-regression						
	No. of studies	Coef. (95% CI)	Std. Err.	Z	*P-*value	Residual I^2^	R^2^ (%)
Study year	17	-0.08 (-0.24, 0.08)	0.08	-1.00	0.315	87.96%	0.00
Study country	17	-0.03 (-0.10, 0.14)	0.06	0.45	0.654	89.00%	0.00
Disease severity	17	0.03 (-0.07, 0.13)	0.05	0.62	0.535	87.60%	0.00
Age	17	0.01 (-0.01, 0.03)	0.00	1.35	0.179	85.84%	0.00
Sex	17	0.00 (-0.01, 0.01)	0.00	0.23	0.821	87.34%	0.00
Hypertension	16	0.00 (-0.01, 0.01)	0.00	-0.50	0.619	89.29%	0.00
Diabetes	16	0.00 (-0.01, 0.01)	0.00	-0.19	0.849	89.02%	0.00
Cortisone use in the IVIg group	13	-0.03 (-0.39, 0.33)	0.18	-0.17	0.865	88.85%	0.00
Cortisone use in the control group	13	0.05 (-0.31, 0.42)	0.18	0.28	0.777	88.63%	0.00
IVIg daily dosage	14	-0.34 (-0.57, -0.11)	0.12	-2.86	**0.004**	72.72%	55.55
IVIg duration	13	-0.06 (-0.12, 0.01)	0.03	-1.76	**0.079**	78.87%	28.86
**Modulators**	**Multivariable meta-regression**						
	**No. of studies**	**Coef. (95% CI)**	**Std. Err.**	**Z**	**p**	**Residual I^2^**	**R^2^ (%)**
IVIg daily dosage	12	-0.25 (-0.48, -0.02)	0.12	-2.15	**0.031**	63.08%	66.71
IVIg duration	12	-0.04 (-0.09, 0.02)	0.03	-1.28	0.199	63.08%	66.71

No., number; Coef., coefficient; CI, confidence interval; Std. Err., standard error; R^2^, the ratio of explained variance to total variance. Bold values were statistically significant values with P-value< 0.05.

**Figure 3:**
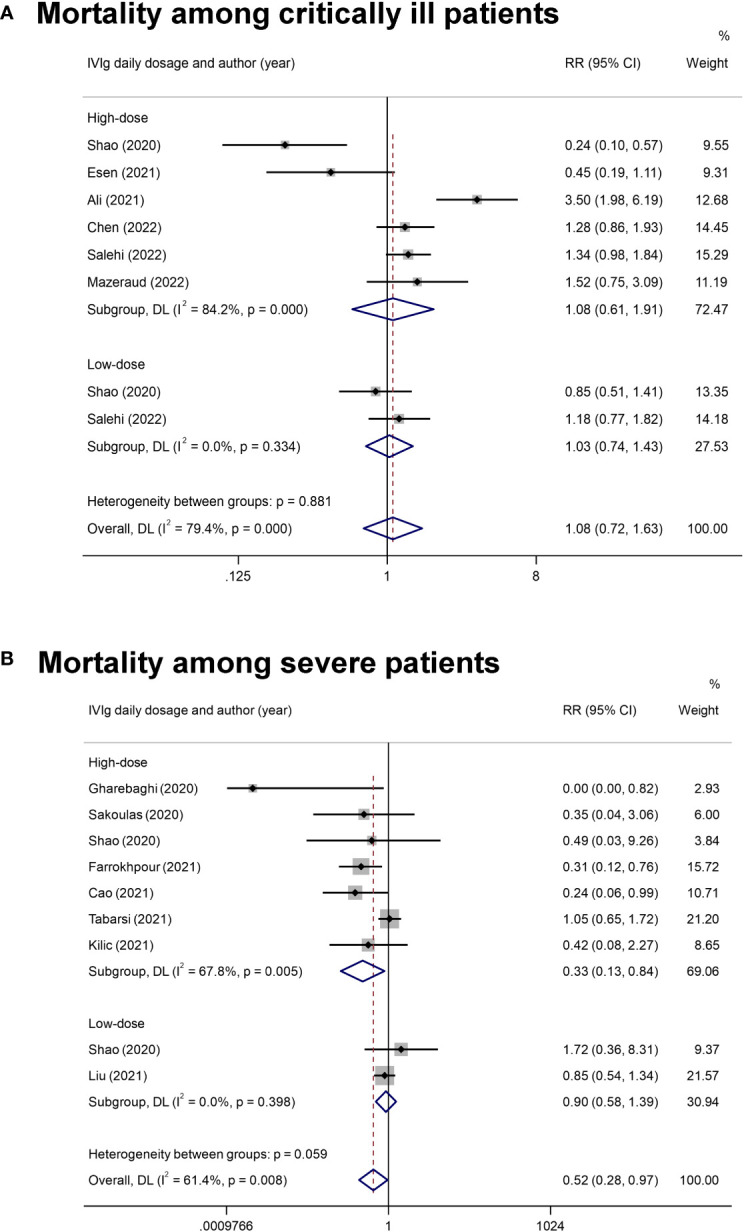
The forest plot of subgroup analyses on IVIg daily dosage. **(A)**, The mortality rate in critically ill COVID-19 patients with the classification into high-dose IVIg group and the low-dose IVIg group. **(B)**, The mortality rate in severe COVID-19 patients with the classification into the high-dose IVIg group and the low-dose IVIg group.

Subgroup analysis focusing on the impact of IVIg daily dosage on the mortality of patients with different disease severity was conducted. Three studies were not been included in the subgroup analysis for lacking a report on the specific dosage and the relevant mortality (43, 46, 49). Results showed that the overall mortality among patients with severe COVID-19 was reduced in high-dose IVIg subgroup (RR= 0.33 [0.13, 0.86], *P*= 0.02, I^2^ = 68%; Very low certainty) (**Figure 3**). Additional analyses were conducted based on the differentially reported adjusted RR from four studies (44, 45, 47, 49), with similar results (**Figure S5**). Interestingly, we noticed that severe patients treated with high-dose IVIg group did not have a longer length of hospital stay (MD = 1.54 [-1.34, 4.42] days, P= 0.29; I^2^ = 80%) (**Figure S6**). The prolonged hospital stay might be associated with the application of inadequate IVIg (**Figure S6**). Further TSA analysis showed that the cumulative Z curve of estimate effects crossed neither the futility, nor the TSA boundaries, it also not exceeded the required information size (**Figures S7, 8**).

### 3.5 Risk of bias assessment

The overall methodological judgments of RCT studies were conducted with ROB 2.0 tool (**Tables S3, S4**). Only one study was considered with a low risk of bias (22), while the rest four studies were considered with a high risk of bias due to the open-labeled design (18, 19, 41, 42). The NOS scores of cohort studies were ranging from 4 to 7, with an average of 5.8 (**Table S5**). Four studies were considered with a low risk of bias (43–45, 47), and the others were considered with the high risk of bias. The further ROBINS-I analysis showed that three studies were judged to be at moderate risk of overall bias (44, 48, 49), while the rest were considered with a serious risk of bias (**Tables S5, S6**). The funnel plots and results from Egger’s regression test showed that no significant publication bias was observed (intercept= 0.90 [-2.68, 0.88], P= 0.30) (**Figure S9**).

## 4 Discussion

This systemic review and meta-analysis of 17 studies involving 4,711 hospitalized COVID-19 patients demonstrated an association between high-dose IVIg therapy and a decreased risk for mortality in severe COVID-19 patients (RR, 0.33; 95%CI, 0.13-0.86; very low certainty). We also found that hospital stay was longer among the IVIg-treated severe COVID-19 patients (MD, 2.62; 95%CI, 0.25-4.99; low certainty). These effects weren’t observed in the latest meta-analysis studies, providing additional evidence that IVIg therapy has implications for the treatment of COVID-19 at different disease severity and dosage.

The dosage and timing of IVIg therapy affected its effects. Study showed that high-dose IVIg significantly reduced mortality compared with the low-dose group (20). Results from other studies also suggested the early administration of IVIg in treating patients with COVID-19 (44, 50, 53, 54) or patients with septic shock (55). For COVID-19 patients who already progressed to critically ill status, the high-dose IVIg was ineffective, indicating its short therapeutic window (48). The major challenge in the application of high-dose IVIg or other immunomodulators is to grasp the proper therapeutic window of the hyper-inflammatory stage of SARS-CoV-2 infection (5, 56–58). Although it is a costly and scarcely available product, the application of high-dose IVIg therapy might be effective among severe COVID-19 patients in clinical prognosis and in the pharmacoeconomic perspective (59).

A previous study reported IVIg could increase the hospital stay in critical subgroup COVID-19 patients (27). Here, we found that using IVIg would prolong the hospital stay of severe COVID-19 patients rather than the critically ill type. We also noticed that the prolonged hospital stay might be associated with the application of inadequate IVIg. However, only one study was included in the subgroup analysis, and more clinical evidence is needed to draw clear conclusions. Besides these, we speculated that the longer duration of hospital stay in IVIg group might be associated with discharge criteria. The IVIg therapy was previously shown to associate with the prolonged duration of SARS-CoV-2 shedding in retrospective cohort studies (60, 61). There were four studies among the 17 included studies that mentioned the outcome. Three studies reported no difference in the duration of the viral clearance between the IVIg group and the control group (43, 44, 50), and interestingly, one study reported a shortened duration of viral clearance in the IVIg group (42). These inconsistencies might be due to the disparity in corticosteroid use of participants among different studies (62, 63). However, further analyses were limited by lacking relevant data, and future work should address these unsolved issues.

Of note, we only included studies using the standard IVIg products. Apart from those, the clinical efficacy of hyperimmune IVIg (hIVIg) and IgM-enriched IVIg (IVIgGM) were also explored by other studies. Different from regular IVIg, the hIVIg products contained a high titer of SARS-CoV-2-specific IgG which could neutralize the virus (64). In a small sample size phase I/II RCT study, the application of hIVIg showed promising effects on improving survival and reducing disease progression (65). However, the recently published international phase III RCT study demonstrated no significant efficacy of hIVIg when compared with the standard care (66). Compared to standard IVIg products which contain more than 96% of IgG with trace amounts of IgM and IgA, the composition of IVIgGM products was similar to the human plasma (38 g/L of IgG, 6 g/L of IgM, and 6 g/L of IgA) (67). Although the IVIgGM therapy had shown more benefit than regular IVIg in treating adult patients with sepsis, its clinical application in treating COVID-19 patients was limited (23, 24, 67–69).

This systematic review and meta-analysis have some limitations. First, the main limitation of the present work is the heterogenicity of the data analyzed. This is noted in the I^2^, which is often higher than 70%. This limits the capability of this kind of analysis to get a firm conclusion. Hence, the reported effect of high-dose IVIg in severe COVID-19 patients was with very low certainty. Besides, the TSA analysis showed that the meta-analysis results might yield false positive and more sample sizes were needed to obtain relatively reliable results. We noticed that many registered clinical trials haven’t published their progress or data. The impact of high-dose IVIg will be further examined after new clinical data become available in the future.

Second, we included 12 cohort studies to pool the treatment effects of IVIg, which raised concerns about the dichotomy definition between severe and critical types. The severity is not very well appraised in retrospective cohort studies as the timing of IVIg administration is not clearly identified. Despite we applied the adjusted effect estimates to pool the relative risk, not all the included studies went through an adequate confounding adjustment. Also, different studies adopted different adjustment methods, affecting the overall combined effects by confounding. Besides, the results may not be generalizable due to the different definitions of disease severity. More studies with restricted designs are needed to address these questions.

Third, limited by the inconsistent report of outcome data from included studies, we only examined the effects of IVIg therapy on mortality, the length of hospital stays, the need for mechanical ventilation, and the incidence of AEs and SAEs. Besides these, other outcomes, such as the duration of mechanical ventilation/ECMO, time to inflammatory factors normalization, and time to viral clearance, would help comprehensively evaluate IVIg’s clinical benefits in treating COVID-19 patients. The effects of high-dose IVIg in regulating immune response could be measured by monitoring the dynamic of proinflammatory cytokines and biomarkers during treatment, which was only reported by a limited number of studies (21, 22, 44).

Last, the IVIg preparations, including regular, IgM-enriched, and SARS-CoV-2 specific ones, were potentially associated with various adverse events (24, 70). Although it was reported that most IVIg-associated adverse events were mild and transient, and occurred in less than 10% of patients, the risk of thromboembolic events requires attention, since both COVID-19 and IVIg might predispose to such SAE (71, 72). Our meta-analysis showed similar incidences in overall AE and SAE between the IVIg group and the control group. However, there was a trend toward a higher incidence of SAE and thromboembolism in the IVIg group of the recently published RCT study (22). Hence, the risks of IVIg-specific adverse events, such as immunologic hemolysis, should be considered in clinical practice.

In summary, by combining data from multiple studies, this systematic review and meta-analysis found that IVIg did not provide significant improvement in mortality, the length of hospital stays, or the use of mechanical ventilation among overall COVID-19 patients. Nevertheless, high-dose IVIg might reduce the mortality in patients with severe COVID-19. However, in combination of the low quality of certainty due to the limited number of studies and the high risk in methodological heterogeneity, the results should be interpreted with great caution, and more research is needed to understand its specific effects. Future research is needed to integrate more well-designed and large-sample clinical trials, and identify the proper dosage and timing of IVIg therapy in treating severe COVID-19 patients.

## Data availability statement

The original contributions presented in the study are included in the article/**Supplementary Material**. Further inquiries can be directed to the corresponding author.

## Author contributions

XSL: Conceptualization, Methodology, Software, Formal analysis, Investigation, Data Curation, Writing - Original Draft, Visualization. YZ: Results interpretation, Writing - Review and Editing. LL: Resources, Data Curation. XDL: Resources, Data Curation. YW: Formal analysis. YY: Formal analysis. WC: Conceptualization, Writing - Review and Editing, Project administration. TL: Conceptualization, Writing - Review and Editing, Supervision, Funding acquisition. All authors contributed to the article and approved the submitted version.
