# Supplementary material for: Benefits of high-dose intravenous immunoglobulin on mortality in patients with severe COVID-19: An updated systematic review and meta-analysis
Source: Front Immunol. 2023 Jan 23;14:1116738. doi: 10.3389/fimmu.2023.1116738 (PMC9900022; doi:10.3389/fimmu.2023.1116738)
Supplement: Supplementary file 1 [file DataSheet_1.docx]

**Supplementary Figures and Tables**

**Authors:** Xiaosheng Liu^1,2,3^, Yuelun Zhang^4^, Lianfeng Lu^3^, Xiaodi Li^3^, Yuanni Wu^3^, Yang Yang^3^, Taisheng Li ^1,3,5^, Wei Cao^3,5*^

Figure S1. The forest plot of mortality with differentially reported adjusted RR.

Figure S2. The forest plot of the length of hospital stay.

Figure S3. The forest plot of the need for mechanical ventilation.

Figure S4. The forest plot of the incidence of adverse events and serious adverse events.

Figure S5. The forest plot of subgroup analyses on IVIg daily dosage with differentially reported adjusted RR.

Figure S6. The trial series analysis (TSA) result on all included 17 studies.

Figure S7. The trial series analysis (TSA) result on severe patients treated with high-dose IVIg.

Figure S8. The funnel plot of publishment bias with mortality rate.

Table S1. Details of protocol changes

Table S2. The additional characteristics of included studies for meta-analysis

Table S3. The risk of bias summary of included RCT studies.

Table S4. The detailed judgements of RCT studies with the RoB 2 assessment tool.

Table S5. The risk of bias summary of included NRSI studies (part 1)

Table S5. The risk of bias summary of included NRSI studies (part 2)

Table S6. The detailed judgements of NRSI studies with the ROBINS-I assessment tool.

**Figure S1. The forest plot of mortality with differentially reported adjusted RR.**

(a), All-cause mortality in COVID-19 patients from included randomized controlled trial (RCT) and cohort studies. (b), The mortality in COVID-19 patients with different disease severity.

**Figure S2. The forest plot of the length of hospital stay.**

(**a**), The length of hospital stay in COVID-19 patients from included randomized controlled trial (RCT) and non-randomized studies on the effects of intervention (NRSI) studies. (**b**), The length of hospital stay in COVID-19 patients with different disease severity. For the RCT study, the risk of bias was assessed in 5 degrees: A, Risk of bias arising from the randomization process; B, Risk of bias due to deviations from the intended interventions; C, Risk of bias due to missing outcome data; D, Risk of bias in measurement of the outcome; E, Risk of bias in selection of the reported result. Red presented for high risk of bias, yellow presented for some concerns, and green presented for low risk of bias. For the NRSI study, the risk of bias was assessed in 7 degrees: A, Bias due to confounding; B, Bias in selection of participants into the study; C, Bias in classification of interventions; D, Bias due to deviations from intended interventions; E, Bias due to missing data; F, Bias in measurement of outcomes; G, Bias in selection of the reported result. Red presented for serious risk of bias, yellow presented for moderate risk of bias, and green presented for low risk of bias.

**Figure S3. The forest plot of the need for mechanical ventilation.**

(**a**), The need for mechanical ventilation in COVID-19 patients from included randomized controlled trail (RCT) and non-randomized studies on the effects of intervention (NRSI) studies. (**b**), The need for mechanical ventilation in COVID-19 patients with different disease severity. For the RCT study, the risk of bias was assessed in 5 degrees: A, Risk of bias arising from the randomization process; B, Risk of bias due to deviations from the intended interventions; C, Risk of bias due to missing outcome data; D, Risk of bias in measurement of the outcome; E, Risk of bias in selection of the reported result. Red presented for high risk of bias, yellow presented for some concerns, and green presented for low risk of bias. For the NRSI study, the risk of bias was assessed in 7 degrees: A, Bias due to confounding; B, Bias in selection of participants into the study; C, Bias in classification of interventions; D, Bias due to deviations from intended interventions; E, Bias due to missing data; F, Bias in measurement of outcomes; G, Bias in selection of the reported result. Red presented for serious risk of bias, yellow presented for moderate risk of bias, and green presented for low risk of bias.

**Figure S4. The forest plot of the incidence of adverse events and serious adverse events.**

For the RCT study, the risk of bias was assessed in 5 degrees: A, Risk of bias arising from the randomization process; B, Risk of bias due to deviations from the intended interventions; C, Risk of bias due to missing outcome data; D, Risk of bias in measurement of the outcome; E, Risk of bias in selection of the reported result. Red presented for high risk of bias, yellow presented for some concerns, and green presented for low risk of bias. For the NRSI study, the risk of bias was assessed in 7 degrees: A, Bias due to confounding; B, Bias in selection of participants into the study; C, Bias in classification of interventions; D, Bias due to deviations from intended interventions; E, Bias due to missing data; F, Bias in measurement of outcomes; G, Bias in selection of the reported result. Red presented for serious risk of bias, yellow presented for moderate risk of bias, and green presented for low risk of bias.

**Figure S5. The forest plot of subgroup analyses on IVIg daily dosage with differentially reported adjusted RR.**

(a), The mortality rate in critically ill COVID-19 patients with the classification into high-dose IVIg group and the low-dose IVIg group. (b), The mortality rate in severe COVID-19 patients with the classification into the high-dose IVIg group and the low-dose IVIg group.

**Figure S6. The forest plot of the length of hospital stay, subgroup analysis based on IVIg daily dosage.**


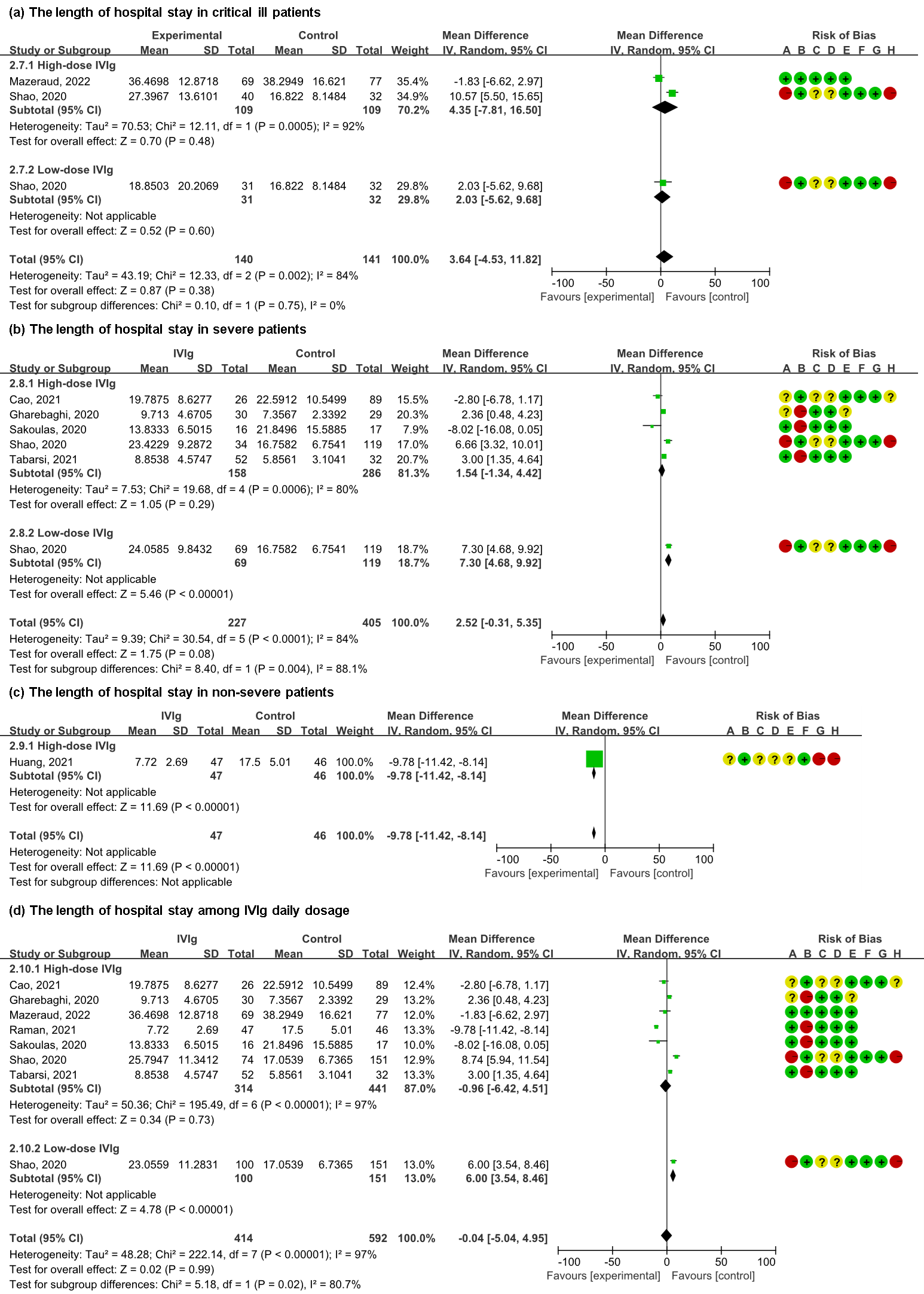


(**a**), The length of hospital stay in critical ill COVID-19 patients with different IVIg daily dosage. (**b**), The length of hospital stay in severe COVID-19 patients with different IVIg daily dosage. (**c**), The length of hospital stay in non-severe COVID-19 patients with different IVIg daily dosage. (**d**), The length of hospital stay in COVID-19 patients with different IVIg daily dosage. For the RCT study, the risk of bias was assessed in 5 degrees: A, Risk of bias arising from the randomization process; B, Risk of bias due to deviations from the intended interventions; C, Risk of bias due to missing outcome data; D, Risk of bias in measurement of the outcome; E, Risk of bias in selection of the reported result. Red presented for high risk of bias, yellow presented for some concerns, and green presented for low risk of bias. For the NRSI study, the risk of bias was assessed in 7 degrees: A, Bias due to confounding; B, Bias in selection of participants into the study; C, Bias in classification of interventions; D, Bias due to deviations from intended interventions; E, Bias due to missing data; F, Bias in measurement of outcomes; G, Bias in selection of the reported result. Red presented for serious risk of bias, yellow presented for moderate risk of bias, and green presented for low risk of bias.

**Figure S7. The trial series analysis (TSA) result on all included 17 studies.**

The type 1 error and power were defined as 5.0% and 80.0). The incidence in control arm and the estimated relative risk reduction were defined as 30.0% and 15.0%.

**Figure S8. The trial series analysis (TSA) result on severe patients treated with high-dose IVIg.**

The type 1 error and power were defined as 5.0% and 80.0). The incidence in control arm and the estimated relative risk reduction were defined as 30.0% and 15.0%.

**Figure S9. The funnel plot of publishment bias with mortality rate.**

**Table S1. Details of protocol changes**

| **Statements of changes** |  |  |  |
| --- | --- | --- | --- |
| The final analysis procedure was adapted from the published PROSPERO protocol in the following ways: (1) Apart from prospective observational studies, retrospective studies were also included in this review; (2) Other outcomes (i.e., time to symptom improvement, duration time of specific treatment modalities, time to inflammatory factors normalization, the duration of positive RT-PCR results) were inconsistently reported by limited studies, hence we only reported the mortality, the length of hospital stay, the needs for mechanical ventilation, the incidence of AEs and SAEs in this review; (3) Instead of using RoB-1 and JBI tools, we applied RoB 2.0 and ROBINS-I tools to assess the risk of bias. | | | |

**Table S2. The additional characteristics of included studies for meta-analysis**

| **First author, Year** | **Age, years** | **Gender (male %)** | **Hypertension (%)** | **Diabetes (%)** |
| --- | --- | --- | --- | --- |
| Gharebaghi, 2020 | 56 (46-62)^a^ | 69.5% | 22.0% | 27.1% |
| Sakoulas, 2020 | 54^b^ | 60.6% | 33.3% | 36.4% |
| Tabarsi, 2021 | 53.6 ± 13.5^c^ | 77.4% | 20.2% | 21.4% |
| Raman, 2021 | 48.7 ± 12.6^c^ | 33.0% | 31.0% | 27.0% |
| Mazeraud, 2022 | 65.8 ± 10.8^c^ | 70.5% | N.A. | N.A. |
| Shao, 2020 | 58 (46–69)^a^ | 58.2% | 30.1% | 11.7% |
| Esen, 2021 | 68.0 ± 15.7^c^ | 73.1% | 24.2% | 14.4% |
| Farrokhpour, 2021 | 69.7 ± 15.4^c^ | 78.8% | 40.9% | 34.8% |
| Cao, 2021 | 59 (47–69)^a^ | 66.1% | 45.2% | 20.9% |
| Huang, 2021 | 37 (25–53)^a^ | 54.3% | 34.1% | 12.6% |
| Liu, 2021 | 63 (55–73)^a^ | 58.9% | 33.5% | 14.9% |
| Hou, 2021 | 55.1 ± 14.2^c^ | 54.0% | 23.0% | 11.5% |
| Ali, 2021 | 53 (42–62)^a^ | 94.1% | 58.5% | 53.0% |
| Kilic, 2021 | 60 (53-69)^a^ | 69.2% | 46.2% | 26.9% |
| Chen, 2022 | 63.8 ± 13.1^c^ | 64.3% | 42.3% | 19.2% |
| Salehi, 2022 | 62.7 ± 15.5^c^ | 59.6% | 42.6% | 31.1% |
| Aggarwal, 2022 | 64 (55–71)^a^ | 75.0% | 55.5% | 52.3% |

Abbreviations: N.A., not available.

a, Data were represented as median and the interquartile range (IQR).

b, Data were represented as mean.

c, Data were represented as mean and the standard deviation (SD).

**Table S3. The risk of bias summary of included RCT studies.**

|  | Gharebaghi, 2020 | Sakoulas, 2020 | Tabarsi, 2021 | Raman, 2021 | Mazeraud, 2022 |
| --- | --- | --- | --- | --- | --- |
| **RoB 2.0** |  |  |  |  |  |
| 1. Risk of bias arising from the randomization process | **Some concerns** | **Low risk** | **Low risk** | **Low risk** | **Low risk** |
| 1. Risk of bias due to deviations from the intended interventions | **High risk** | **High risk** | **High risk** | **High risk** | **Low risk** |
| 1. Risk of bias due to missing outcome data | **Low risk** | **Low risk** | **Low risk** | **Low risk** | **Low risk** |
| 1. Risk of bias in measurement of the outcome | **Low risk** | **Low risk** | **Low risk** | **Low risk** | **Low risk** |
| 1. Risk of bias in selection of the reported result | **Some concerns** | **Low risk** | **Low risk** | **Low risk** | **Low risk** |
| **Overall risk** | **High risk** | **High risk** | **High risk** | **High risk** | **Low risk** |
| **mJADAD Scale** |  |  |  |  |  |
| 1. Was the method of randomization appropriate? | yes | yes | yes | yes | yes |
| 1. Was the study described as blinded? (double-blind with score 1; single-blind with score 0.5) | yes | no | no | no | yes |
| 1. Was the method of blinding appropriate? | yes | no | no | no | yes |
| 1. Was there a description of withdrawals and dropouts? | no | no | no | no | yes |
| 1. Was there a clear description of the inclusion/exclusion criteria? | no | no | yes | yes | yes |
| 1. Was the method used to assess adverse effects described? | no | yes | yes | yes | yes |
| 1. Was the method of statistical analysis described? | yes | yes | yes | yes | yes |
| **Total Scores** | 5 | 4 | 5 | 5 | 8 |

**Table S4. The detailed judgements of RCT studies with the RoB 2 assessment tool.**

**Gharebaghi, 2020**

| **Methods** | **A randomized placebo-controlled double-blind clinical trial.** |
| --- | --- |
| **Participants** | Severe COVID-19 who did not respond to initial treatments from an undisclosed teaching hospital in Iran. The name of the teaching hospital was hidden due to blind peer-review protocol. Patients consecutively were recruited between May 9th, 2020 and June 9th, 2020. Inadequate response to initial treatment was defined as the lack of improvement of dyspnea, fever, and hypoxemia (satO2 less than 90%), as well as the need for oxygenation to maintain satO2 above 90% after 48 h of commencing treatment. |
| **Experimental** | The IVIg group received IVIg (human) flebogamma 5% DIF GRIFOLS, in addition to their prior initial treatment (the initial treatment methods continued in the treatment group during the trial). Treatment group patients received four vials of 5 gm5 IVIg daily for three consecutive days. |
| **Comparator** | The control group continued to receive the same treatments as were introduced initially, in addition to a placebo. Placebo and IVIg vials were similar in appearance and contained a similar volume of solution. Placebo vials contained saline solution. |
| **Outcomes** | The outcome didn't list along in Methods, the authors majorly reported the In-hospital mortality rate. |
| **Results** | Among total study subjects, 30 patients received IVIg and 29 patients received a placebo. Demographics, clinical characteristics, and laboratory tests were not statistically different (P > 0.05) between the two groups. The in-hospital mortality rate was significantly lower in the IVIg group compared to the control group (6 [20.0%] vs. 14 [48.3%], respectively; P = 0.022). Multivariate regression analysis demonstrated that administration of IVIg did indeed have a significant impact on mortality rate (aOR = 0.003 [95% CI: 0.001–0.815]; P = 0.042). |

**Risk of bias table**

| **Bias** | **Support for judgement** | **Authors' judgement** |
| --- | --- | --- |
| **Risk of bias arising from the randomization process** | Study subjects were randomly assigned into two approximately equal groups: IVIg treatment and placebo control using a computer-generated randomization schedule. Demographics, clinical characteristics, and evaluated laboratory tests between the treatment and control group did not exhibit significant differences except for between: the (1) serum creatinine (mg/dl), which was higher in the control group (1.0 [0.8–1.1] in the treatment group vs. 1.2 [1.0–1.4] in the control group; P=0.001), (2) white blood cell (WBC) count (1000/mm3), which was also higher in the control group (5.05 [4.20–7.00] in the treatment group vs. 6.60 [5.00–10.90] in the control group; P=0.026), and (3) the overall duration of hospitalization (days) was longer in the treatment group (9 [7–13] in the treatment group vs. 7 [6–9] in the control group; P=0.014), these difference suggested some concerns in the risk of bias of randomization. | **Some concerns** |
| **Risk of bias due to deviations from the intended interventions** | Neither patients nor physicians nor data analysts were aware of treatment versus placebo membership. The only individual that did was the pharmacist of the study center. However, patients who died before 72h after the distribution of IVIg and placebos were excluded from this study due to an incomplete course of treatment. Only per-protocol analysis results were provided, with the missing report of the intention-to-treat population. There might be potential for a substantial impact (on the result) of the failure to analyze participants in the group to which they were randomized. | **High risk** |
| **Risk of bias due to missing outcome data** | Data for this outcome available for all participants randomized were provided. | **Low risk** |
| **Risk of bias in measurement of the outcome** | The method of measuring the outcome was appropriate. And the risk in measurement or ascertainment of the outcome to have differed between intervention groups was low. | **Low risk** |
| **Risk of bias in selection of the reported result** | This study protocol was preregistered in https://www.irct.ir/trial/47609. The original primary outcome was increasing of patient's O2 saturation above 90%, improvement of lung involvement in lung CT scan, with empty records in secondary outcomes. However, the published study majorly discussed the In-hospital mortality rate. The data that produced this result analyzed did not in accordance with a pre-specified analysis plan that was finalized before unblinded outcome data were available for analysis. | **Some concerns** |
| **Overall risk of bias** |  | **High risk** |

**Sakoulas, 2020**

| **Methods** | **A Prospective randomized open label clinical trial.** |
| --- | --- |
| **Participants** | Severe COVID-19 from Sharp Memorial Hospital (San Diego, CA) and Sharp Grossmont Hospital (La Mesa, CA) were considered for inclusion if they demonstrated moderate-to-severe hypoxia (sPo2 ≤ 96% on ≥ 4 L O2 by nasal cannula) but not on mechanical ventilation. Patients were considered for enrollment when treating hospitalists and/or intensivists notified the study team for consideration. |
| **Experimental** | The IVIG treatment arm consisted of the subject receiving IVIG (Octagam 10% provided by Octapharma USA) 0.5-g/kg IV daily for 3 days beginning on the day of enrollment, in addition to SOC. For subjects not already receiving glucocorticoid therapy, enrolled treatment arm subjects received methylprednisolone 40-mg IV once 30 minutes before IVIG to mitigate headache commonly experienced after IVIG therapy. |
| **Comparator** | Standard of care (SOC) treatment as control. SOC consisted of the subject remaining on or being eligible for any treatment (e.g., glucocorticoids, convalescent plasma, and remdesivir) not part of a randomized clinical trial. |
| **Outcomes** | The subject was enrolled and monitored for progression to the endpoint of: 1) respiratory failure requiring receipt of mechanical ventilation (a composite of either receiving ventilation or the subject status changed to a do not resuscitate/do not intubate resulting in progressive respiratory failure and death) or 2) death from no respiratory causes prior to receipt of mechanical ventilation. If the subject progressed to mechanical ventilation, receipt of off-label agents and/or enrollment in other clinical trials was allowed. Subject hospital course was followed until hospital discharge for the purpose of total and ICU days of hospital days. |
| **Results** | Sixteen subjects received IV immunoglobulin and 17 standards of care. Median ages were 51 and 58 years for the standard of care and IV immunoglobulin, respectively. Acute Physiology and Chronic Health Evaluation II and Charlson comorbidity scores were similar for IV immunoglobulin and standard of care. Seven standards of care versus two IV immunoglobulin subjects required mechanical ventilation (p = 0.12, Fisher exact test). Among subjects with A-a gradient of greater than 200 mm Hg at enrollment, the IV immunoglobulin group showed: 1) a lower rate of progression to requiring mechanical ventilation (2/14 vs 7/12, p = 0.038 Fisher exact test), 2) shorter median hospital length of stay (11 vs 19 d, p = 0.01 Mann Whitney U test), 3) shorter median ICU stay (2.5 vs 12.5 d, p = 0.006 Mann Whitey U test), and 4) greater improvement in Pao2/Fio2 at 7 days (median [range] change from time of enrollment +131 [+35 to +330] vs +44·5 [–115 to +157], p = 0.01, Mann Whitney U test) than standard of care. Pao2/Fio2 improvement at day 7 was significantly less for the standard of care patients who received glucocorticoid therapy than those in the IV immunoglobulin arm (p = 0.0057, Mann Whiney U test). |

**Risk of bias table**

| **Bias** | **Support for judgement** | **Authors' judgement** |
| --- | --- | --- |
| **Risk of bias arising from the randomization process** | After informed consent was obtained electronically by DocuSign (San Francisco, CA), subjects were randomized 1:1 into the treatment arm or standard of care (SOC) control arm. Author M.G., the randomizing investigator, used web-based computer-generated randomization (A study [IVIG] group, B control [SOC] group) in blocks of 10 (https://www.sealedenvelope.com/simple-randomiser/v1/lists). When the randomization list was generated, M.G. placed the codes into individual sealed and sequentially numbered envelopes. The batch of sealed envelopes was stacked in sequential order and retained in a locked drawer in the Investigational Research Pharmacy. After informed consent was obtained from a potential study subject, M.G. obtained the next numbered envelope (i.e., #001, #002, #003, etc.) to obtain the A, B randomization code for treatment arm allocation. Of note, the subjects were well matched with respect to age, underlying comorbidities, concomitant therapies and laboratory characteristics, indicating the low risk of bias in randomization. | **Low risk** |
| **Risk of bias due to deviations from the intended interventions** | This study was conducted in open-label. Participants, carers, and people delivering the interventions would be aware of participants' assigned intervention during the trial. There was no information to determine whether deviations from the intended intervention arose because of the trial context. Of note, immediately after randomization and notification of the principal investigator, one subject was immediately deemed unevaluable by the principal investigator and excluded due to a high risk of bacterial superinfection (elevated absolute neutrophil count of 9,900/mm3 and procalcitonin of 1.45 ng/mL). The rest of included patients were analyzed without any exclusion. | **High risk** |
| **Risk of bias due to missing outcome data** | Data for this outcome available for all participants randomized were provided. | **Low risk** |
| **Risk of bias in measurement of the outcome** | The method of measuring the outcome was appropriate. And the risk in measurement or ascertainment of the outcome to have differed between intervention groups was low. | **Low risk** |
| **Risk of bias in selection of the reported result** | This study protocol was preregistered in https://clinicaltrials.gov/ct2/show/NCT04411667. The study outcome and analysis methods were in accordance with the pre-specified analysis plan that was finalized before unblinded outcome data were available for analysis. | **Low risk** |
| **Overall risk of bias** |  | **High risk** |

**Tabarsi, 2021**

| **Methods** | **A Prospective randomized open label clinical trial.** |
| --- | --- |
| **Participants** | Severe ill adult COVID-19 patients from Dr. Masih Daneshvari Hospital, a university-affiliated and selected referral center for COVID-19 patients, Tehran, Iran were considered for inclusion if they demonstrated moderate-to-severe hypoxia (sPo2 ≤ 96% on ≥ 4 L O2 by nasal cannula) but not on mechanical ventilation. Patients were considered for enrollment when treating hospitalists and/or intensivists notified the study team for consideration. |
| **Experimental** | Patients in the IVIg group received Intratect® (Biotest), 400 mg/Kg daily for three doses if they met the necessary criteria for receiving the drug. All patients in the IVIg group were premedicated with 500 mg Acetaminophen, 100 mg Hydrocortisone, and 25 mg Diphenhydramine 30 min before the injection. |
| **Comparator** | Patients in both groups received oxygen and fluid support, lopinavir/ritonavir (200/50 mg, Hetero labs), two tablets twice a day, and hydroxychloroquine (Tehran-Daru) 200 mg two times daily. |
| **Outcomes** | The primary outcomes included the need for invasive mechanical ventilation and oxygenation, the need for admission to the Intensive Care Unit (ICU), and the mortality rate. Secondary outcomes included length of stay in ICU and hospital, and radiological improvements in the CT scan. The data were collected from the patients’ medical records, which included age, gender, underlying diseases, laboratory test results, and CT imaging findings. |
| **Results** | The mean time from admission to IVIg initiation was 3.84 ± 3.35 days. There was no significant difference between the two groups in terms of mortality rate (P-value = 0.8) and the need for mechanical ventilation (P-value = 0.39). The length of hospital stay was significantly lower for the control group than that of the intervention group (P-value = 0.003). There was a significant positive relationship between the time from hospital admission to IVIg initiation and the length of stay in the hospital and ICU among the survivors (P-value < 0.001 and =0.01, respectively). |

**Risk of bias table**

| **Bias** | **Support for judgement** | **Authors' judgement** |
| --- | --- | --- |
| **Risk of bias arising from the randomization process** | Block randomization method was used for randomization. Eight blocks, including ten patients, were generated by the Online Randomizer website (www.sealesenvelop.com/simple randomizer). There were no significant differences between the two groups in terms of patients’ age, gender, and past medical history, indicating the low risk of bias in randomization. | **Low risk** |
| **Risk of bias due to deviations from the intended interventions** | This study was conducted in open-label. Participants, carers, and people delivering the interventions would be aware of participants' assigned intervention during the trial. There was no information to determine whether deviations from the intended intervention arose because of the trial context. All randomized patients were included in the analysis. | **High risk** |
| **Risk of bias due to missing outcome data** | Data for this outcome available for all participants randomized were provided. | **Low risk** |
| **Risk of bias in measurement of the outcome** | The method of measuring the outcome was appropriate. And the risk in measurement or ascertainment of the outcome to have differed between intervention groups was low. | **Low risk** |
| **Risk of bias in selection of the reported result** | This study protocol was preregistered in https://en.irct.ir/trial/49638. The study outcome and analysis methods were in accordance with the pre-specified analysis plan that was finalized before unblinded outcome data were available for analysis. | **Low risk** |
| **Overall risk of bias** |  | **High risk** |

**Raman, 2021**

| **Methods** | **A Prospective randomized open label clinical trial.** |
| --- | --- |
| **Participants** | Moderate COVID-19 patients from 4 centers across four Indian cities, between July 2020 and September 2020 were considered for inclusion. |
| **Experimental** | Patients daily received immunoglobulin 0.4 g/kg bodyweight for 5 days. |
| **Comparator** | Patients in both groups received standard of care (SOC), the SOC consisted of Azithromycin; Lopinavir/ritonavir; Piperacillin + Tazobactam; Acetaminophen and Pantocid. |
| **Outcomes** | The primary endpoint was number of days from initiation of treatment to hospital discharge. The secondary endpoints were: Time taken for improvement of clinical parameters which included  number of days for normalization of body temperature (<37°C), oxygen saturation (>94% on room air) and duration of cough; duration of mechanical ventilation from day 0 to 28; number of deaths during the follow-up of 28 days; proportion of patients with negative RT-PCR during the study period on day 14, on day 28 or end of the study period. |
| **Results** | Duration of hospital stay was significantly shorter in IVIG group to that of SOC alone (7.7 Vs. 17.5 days). Duration for normalization of body temperature, oxygen saturation and mechanical ventilation were significantly shorter in IVIG compared to SOC. Percentages of patients on mechanical ventilation in two groups were not significantly different (24% Vs. 38%). Median time to RT-PCR negativity was significantly shorter with IVIG than SOC (7 Vs.18 days). There were only mild to moderate adverse events in both groups except for one patient (2%), who died in SOC. |

**Risk of bias table**

| **Bias** | **Support for judgement** | **Authors' judgement** |
| --- | --- | --- |
| **Risk of bias arising from the randomization process** | Computer-generated block randomization. There were no statistically significant differences between the patients of the two treatment groups regarding age, gender, Body Mass Index and co-existing co-morbid conditions such as diabetes, hypertension and obesity between the two groups. Besides, there were no significant differences in hematological and biochemical parameters between the two treatment groups at the time of baseline | **Low risk** |
| **Risk of bias due to deviations from the intended interventions** | This study was conducted in open-label. Participants, carers, and people delivering the interventions would be aware of participants' assigned intervention during the trial. There was no information to determine whether deviations from the intended intervention arose because of the trial context. 4 patients were discontinued during follow-up, the rest of the randomized patients were included in the analysis (PP analysis). | **High risk** |
| **Risk of bias due to missing outcome data** | Data for this outcome available for all participants randomized were provided. | **Low risk** |
| **Risk of bias in measurement of the outcome** | The method of measuring the outcome was appropriate. And the risk in measurement or ascertainment of the outcome to have differed between intervention groups was low. | **Low risk** |
| **Risk of bias in selection of the reported result** | This study protocol was preregistered in Clinical Trial Registry, India (CTRI/2020/06/026222). The study outcome and analysis methods were in accordance with the pre-specified analysis plan that was finalized before unblinded outcome data were available for analysis. | **Low risk** |
| **Overall risk of bias** |  | **High risk** |

**Mazeraud, 2022**

| **Methods** | **A randomized placebo-controlled double-blind clinical trial.** |
| --- | --- |
| **Participants** | Critically ill patients (≥18 years) with COVID-19 who admitted to the intensive care unit were eligible for inclusion if they required invasive mechanical ventilation for moderate-to-severe ARDS, according to the Berlin Definition criteria, from 43 centers in France. |
| **Experimental** | In addition to SOC, IVIG was administered for a total dose of 2 g/kg. IVIG infusion had to start before the end of the 96 h after the onset of invasive mechanical ventilation. To minimize the risk of adverse events, IVIG administration was divided into four perfusions of 0·5g/kg each given over at least 8 h over 4 days. |
| **Comparator** | In addition to SOC, patients in the placebo group had to receive an equivalent volume of sodium chloride 0·9% (10 mL/kg), over the same period. |
| **Outcomes** | The primary outcome was the number of ventilator-free days at day 28, defined as the number of days between the last extubation day and day 28. In the case of death before day 28, the score was zero. The primary outcome composite components were as time-to-event censored at day 28, within a competing risk framework; therefore, on day 28, the secondary efficacy endpoints of mortality, the proportion of patients who were extubated, and the duration of invasive mechanical ventilation were collected as subcomponents of the primary endpoint, measured according to the intention-to-treat population. The key secondary outcomes were the sequential organ failure assessment score at day 14 and day 28; the occurrence of grade 3 or 4 adverse events or serious adverse events attributed to IVIG; the time to intensive care unit or hospital discharge; the clinical status at day 28 and day 90 as assessed by the seven-category ordinal scale; 90-day mortality; and lung injury score at day 28.26 The main exploratory secondary outcomes were occurrence of pulmonary embolism and nosocomial pneumonia within the first 28 days, cytokines concentrations (IL-6, TNF-α, and IL-13), and circulating lymphocytes populations at admission, days 7, 14, and 28. All were measured in the intention-to-treat population. Adverse events were coded using the MedDRA (version 22) coding dictionary; each adverse event was electronically reported to the sponsor and monitored until its complete resolution. The number of deaths due to an adverse event and study discontinuation due to an adverse event was recorded. The safety analysis included all patients who received at least one dose of the study drug. |
| **Results** | 146 patients (43 [29%] women) were eligible for inclusion and randomly assigned: 69 (47%) patients to the IVIG group and 77 (53%) to the placebo group. The intention-to-treat analysis showed no statistical difference in the median number of ventilation-free days at day 28 between the IVIG group (0·0 [IQR 0·0–8·0]) and the placebo group (0·0 [0·0–6·0]; difference estimate 0·0 [0·0–0·0]; p=0·21). Serious adverse events were more frequent in the IVIG group (78 events in 22 [32%] patients) than in the placebo group (47 events in 15 [20%] patients; p=0·089). |

**Risk of bias table**

| **Bias** | **Support for judgement** | **Authors' judgement** |
| --- | --- | --- |
| **Risk of bias arising from the randomization process** | Randomization was done with a web-based system. Trial group designation was concealed and the randomization group was electronically sent to the center’s pharmacy. Patients were assigned (1:1) to receive either IVIG (IVIG group) or placebo (placebo group). Random assignment was stratified according to the participating center and the duration of invasive mechanical ventilation before inclusion in the trial (<12 h, 12–24 h, and >24–36 h). The protocol was amended to extend the last temporal category to 72 h. The baseline characteristics between the two groups were similar, indicating the low risk of bias in randomization. | **Low risk** |
| **Risk of bias due to deviations from the intended interventions** | Trial participants, care providers, and outcome assessors were masked to patient assignment. The double-blinding was provided by each hospital pharmacy, using opaque sleeves and tubing to conceal the product administered. To preserve the masking, research nurses supervised the administration and were asked not to disclose whether IVIG or placebo was infused. Masking was removed in the event of an adverse effect that could be attributed to IVIG or placebo and upon the responsible investigator's approval. The statisticians who analyzed the data were masked to group assignment. ITT analysis were conducted, which would reduce the bias due to deviations from the intended interventions. | **Low risk** |
| **Risk of bias due to missing outcome data** | Data for this outcome available for all participants randomized were provided. | **Low risk** |
| **Risk of bias in measurement of the outcome** | The method of measuring the outcome was appropriate. And the risk in measurement or ascertainment of the outcome to have differed between intervention groups was low. | **Low risk** |
| **Risk of bias in selection of the reported result** | This study protocol was preregistered in NCT04350580. The study outcome and analysis methods were in accordance with the pre-specified analysis plan that was finalized before unblinded outcome data were available for analysis. | **Low risk** |
| **Overall risk of bias** |  | **Low risk** |

**Table S5. The risk of bias summary of included NRSI studies (part 1)**

|  | Shao, et al. 2020 | Esen, et al. 2021 | Farrokhpour, et al. 2021 | Cao, et al. 2021 | Huang, et al. 2021 | Liu, et al. 2021 |
| --- | --- | --- | --- | --- | --- | --- |
| **ROBINS-I** |  |  |  |  |  |  |
| 1. Bias due to confounding | **Serious risk** | **Serious risk** | **Serious risk** | **Moderate risk** | **Moderate risk** | **Moderate risk** |
| 1. Bias in selection of participants into the study | **Low risk** | **Moderate risk** | **Moderate risk** | **Low risk** | **Low risk** | **Low risk** |
| 1. Bias in classification of interventions | **Moderate risk** | **Moderate risk** | **Moderate risk** | **Moderate risk** | **Moderate risk** | **Moderate risk** |
| 1. Bias due to deviations from intended interventions | **Moderate risk** | **Moderate risk** | **Moderate risk** | **Moderate risk** | **Moderate risk** | **Moderate risk** |
| 1. Bias due to missing data | **Low risk** | **Low risk** | **Low risk** | **Low risk** | **Moderate risk** | **Moderate risk** |
| 1. Bias in measurement of outcomes | **Low risk** | **Low risk** | **Low risk** | **Low risk** | **Low risk** | **Low risk** |
| 1. Bias in selection of the reported result | **Low risk** | **Low risk** | **Low risk** | **Low risk** | **Serious risk** | **Serious risk** |
| **Overall bias** | **Serious risk** | **Serious risk** | **Serious risk** | **Moderate risk** | **Serious risk** | **Serious risk** |
| **NOS scale** |  |  |  |  |  |  |
| 1. Representativeness of the exposed cohort | 0 | 0 | 0 | 1 | 0 | 0 |
| 1. Selection of the non exposed cohort | 0 | 0 | 0 | 0 | 0 | 1 |
| 1. Ascertainment of exposure | 1 | 1 | 1 | 1 | 1 | 1 |
| 1. Demonstration that outcome of interest was not present at start of study | 1 | 1 | 1 | 1 | 1 | 1 |
| 1. Comparability of cohorts on the basis of the design or analysis | 1 | 1 | 1 | 2 | 2 | 2 |
| 1. Assessment of outcome | 1 | 1 | 1 | 1 | 1 | 1 |
| 1. Was follow-up long enough for outcomes to occur | 1 | 1 | 0 | 0 | 1 | 0 |
| 1. Adequacy of follow up of cohorts | 1 | 1 | 1 | 1 | 1 | 1 |
| **Total Scores** | 6 | 6 | 5 | 7 | 7 | 7 |

The NOS scale includes 8 items in 3 domains, with a total of 9 scores. Studies with scores > 6 can be considered to be of high quality and low risk.

**Table S5. The risk of bias summary of included NRSI studies (part 2)**

|  | Hou, et al. 2021 | Ali, et al. 2021 | Kilic, et al. 2021 | Chen, et al. 2021 | Salehi, et al. 2021 | Aggarwal, et al. 2022 |
| --- | --- | --- | --- | --- | --- | --- |
| **ROBINS-I** |  |  |  |  |  |  |
| 1. Bias due to confounding | **Serious risk** | **Moderate risk** | **Serious risk** | **Moderate risk** | **Low risk** | **Serious risk** |
| 1. Bias in selection of participants into the study | **Low risk** | **Moderate risk** | **Moderate risk** | **Low risk** | **Low risk** | **Moderate risk** |
| 1. Bias in classification of interventions | **Moderate risk** | **Moderate risk** | **Moderate risk** | **Moderate risk** | **Moderate risk** | **Moderate risk** |
| 1. Bias due to deviations from intended interventions | **Moderate risk** | **Moderate risk** | **Moderate risk** | **Moderate risk** | **Low risk** | **Moderate risk** |
| 1. Bias due to missing data | **Low risk** | **Low risk** | **Low risk** | **Low risk** | **Low risk** | **Low risk** |
| 1. Bias in measurement of outcomes | **Low risk** | **Low risk** | **Low risk** | **Low risk** | **Low risk** | **Low risk** |
| 1. Bias in selection of the reported result | **Low risk** | **Serious risk** | **Low risk** | **Low risk** | **Low risk** | **Low risk** |
| **Overall bias** | **Serious risk** | **Serious risk** | **Serious risk** | **Moderate risk** | **Moderate risk** | **Serious risk** |
| **NOS scale** |  |  |  |  |  |  |
| 1. Representativeness of the exposed cohort | 0 | 0 | 0 | 0 | 1 | 0 |
| 1. Selection of the non exposed cohort | 0 | 1 | 0 | 0 | 1 | 0 |
| 1. Ascertainment of exposure | 1 | 1 | 1 | 1 | 1 | 1 |
| 1. Demonstration that outcome of interest was not present at start of study | 1 | 1 | 1 | 1 | 1 | 1 |
| 1. Comparability of cohorts on the basis of the design or analysis | 0 | 2 | 0 | 2 | 0 | 0 |
| 1. Assessment of outcome | 1 | 1 | 1 | 1 | 1 | 1 |
| 1. Was follow-up long enough for outcomes to occur | 0 | 0 | 1 | 0 | 0 | 0 |
| 1. Adequacy of follow up of cohorts | 1 | 1 | 1 | 1 | 1 | 1 |
| **Total Scores** | 4 | 7 | 5 | 6 | 6 | 4 |

The NOS scale includes 8 items in 3 domains, with a total of 9 scores. Studies with scores > 6 can be considered to be of high quality and low risk.

**Table S6. The detailed judgements of NRSI studies with the ROBINS-I assessment tool.**

**Shao, et al. 2020**

| **Methods** | **A multicenter retrospective cohort study.** |
| --- | --- |
| **Participants** | Severe and critical COVID-19 patients who were admitted to 4 government‐designated treatment centres for COVID‐19 patients in 3 cities in China, including Wuhan, Guangzhou and Shenzhen, from December 2019 to March 2020. |
| **Interventions** | IVIg and non-IVIg use during treatment. The doses used differed among the different centres and physicians, ranging from 0.1 to 0.5 g/kg per day for infusion. The treatment period ranged from 5 to 15 days. |
| **Outcomes** | 28‐day mortality and 60‐day mortality as the primary outcomes; In‐hospital days and total course of the disease as the secondary outcomes. |
| **Notes** | The administration dose and timing of IVIg were different (≤15 g per day or >5 g per day; ≤7 days or >7 days), the subgroup analysis and multivariate analysis were conducted to adjust. |

**Risk of bias table**

| **Bias** | **Support for judgement** | **Authors' judgement** |
| --- | --- | --- |
| **Bias due to confounding** | Differences in baseline and clinical characteristics were significant. Comparisons of baseline characteristics between the two groups showed that the disease was more severe in the IVIG group, presented by older age, higher APACH II (Acute Physiology and Chronic Health Evaluation II) scores and SOFA (Sequential Organ Failure Assessment) scores, higher levels of total bilirubin, direct bilirubin, creatinine, C‐reactive protein, IL‐6 and lactate, but lower platelets and lymphocyte count (all P < 0.05), and decreased PaO2/FiO2 (P = 0.011, Table 1). Although the subgroup analysis and multivariate analysis were conducted to adjust, the bias might not be reduced. | **Serious risk** |
| **Bias in selection of participants into the study** | The selection of participants into the study was not based on the participant characteristics observed after the start of the intervention. Among 338 confirmed critical COVID‐19 patients, 325 patients were included in the final analysis after excluding 13 patients because of missing key information. | **Low risk** |
| **Bias in classification of interventions** | The classification and assignment of IVIg or control group were determined retrospectively. | **Moderate risk** |
| **Bias due to deviations from intended interventions** | The treatment difference between the two groups wasn't reported. | **Moderate risk** |
| **Bias due to missing data** | The primary outcomes of all included patients (IVIg, n= 174; Non-IVIg, n=151) were reported. And no participants were excluded due to missing data on intervention status or due to missing data on other variables needed for the analysis. | **Low risk** |
| **Bias in measurement of outcomes** | The outcome assessors might be aware of the intervention received by study participants; however, the outcomes of the study were objectified (28‐day mortality, 60‐day mortality, in‐hospital days and total course of the disease). And the methods of outcome assessment were comparable across intervention groups. | **Low risk** |
| **Bias in selection of the reported result** | The effect estimates were not to be selected reported on the basis of the results, from multiple outcome measurements, multiple analyses of the intervention-outcome relationship, or different subgroups. | **Low risk** |
| **Overall bias** |  | **Serious risk** |

**Esen, et al. 2021**

| **Methods** | **A single-center retrospective cohort study.** |
| --- | --- |
| **Participants** | Critically ill patients who were admitted to two ICU wards of the University Hospital of Istanbul (Turkey), from 19 March to 26 May 2020. |
| **Interventions** | IVIg and non-IVIg use during treatment. IVIG 5% was added at a dose 30 g/day for five consecutive days, on an individual case basis in one of the two ICUs. |
| **Outcomes** | Clinical outcome measures included duration of specific treatment modalities (extracorporeal membrane oxygenation, vasopressors, renal replacement, cytokine adsorption, high-flow nasal cannula and mechanical ventilation), time to start of mechanical ventilation, change in ventilation mode, ICU and hospital discharge, and overall survival. |
| **Notes** | In this study, Kaplan–Meier and multiple logistic regression models were used to analyze the effect of IVIg. |

**Risk of bias table**

| **Bias** | **Support for judgement** | **Authors' judgement** |
| --- | --- | --- |
| **Bias due to confounding** | The IVIG group was younger and had slightly lower baseline disease scores though (Table 1). There were no major differences in concomitant COVID-19 treatments while the distribution of concurrent diseases was different with 1.5 to 2-fold higher prevalence of diabetes and malignancies in the IVIG group and of chronic cardiac, chronic renal and cerebrovascular disease in the SIC group (Table 2). Accordingly, proBNP and troponin levels at baseline were lower in the IVIG group, i.e. 1.02 (interquartile range 0.22 to 2.54) vs. 2.62 (0.32 to 7.48) µg/L and 9.3 (21.1 to 53.0) vs. 17.0 (56.0 to 114.0) ng/L. Although the Kaplan–Meier and multiple logistic regression models were used to analyze the effect of IVIg, bias might not be reduced. | **Serious risk** |
| **Bias in selection of participants into the study** | The selection of participants into the study was not based on the participant characteristics observed after the start of the intervention. However, the select flow on included 93 patients was not shown. | **Moderate risk** |
| **Bias in classification of interventions** | The classification and assignment of IVIg or control group were determined retrospectively. | **Moderate risk** |
| **Bias due to deviations from intended interventions** | The treatment criteria of the two group were specified in the University of Istanbul Directive. However, patients were assigned to each ward by a dedicated gatekeeper physician who decided based on the availability of staff and bedsides. High-dose IVIG was added to SIC in one of two separate wards (in the Department of Internal Medicine) without informing the other (in the Department of Anesthesiology and Intensive Care), i.e. assignment to treatment with or without IVIG eventually was at random. | **Moderate risk** |
| **Bias due to missing data** | The primary outcomes of all included patients (IVIg, n= 51; Non-IVIg, n=42) were reported. And no participants were excluded due to missing data on intervention status or due to missing data on other variables needed for the analysis. | **Low risk** |
| **Bias in measurement of outcomes** | The outcome assessors might be aware of the intervention received by study participants; however, the outcomes of the study were objectified (duration of specific treatment modalities, time to start of mechanical ventilation, change in ventilation mode, ICU and hospital discharge, and overall survival). And the methods of outcome assessment were comparable across intervention groups. | **Low risk** |
| **Bias in selection of the reported result** | The effect estimates were not to be selected reported on the basis of the results, from multiple outcome measurements, multiple analyses of the intervention-outcome relationship, or different subgroups. | **Low risk** |
| **Overall bias** |  | **Serious risk** |

**Farrokhpour, et al. 2021**

| **Methods** | **A single-center retrospective cohort study.** |
| --- | --- |
| **Participants** | Patients who were intubated and admitted to the  ICU of a multispecialty hospital in March 2020 (the first  month of COVID-19 outbreak in Tehran, Iran) were included in this study. |
| **Interventions** | There were four groups in this study: Infliximab, IVIg, infliximab plus IVIg and control. The dosage of IVIg therapy was 400 mg/kg/d slow infusion over 6 hours for 3 to 5 days; and the dosage of infliximab was infusion of 5 mg/kg as a single dose in 350 ml of saline 0.9% serum injected intravenously over 3 to 4 hours slowly. |
| **Outcomes** | Mortality, hospitalization duration and ICU admission duration. |
| **Notes** | In this study, cox regression analysis was conducted and the HR of therapy in mortality was calculated. |

**Risk of bias table**

| **Bias** | **Support for judgement** | **Authors' judgement** |
| --- | --- | --- |
| **Bias due to confounding** | Differences in baseline and clinical characteristics were significant. The patients in the IVIg groups were significantly younger than those in the control groups (64.5±12.9 vs. 72.4±16.0, p = 0.047) | **Serious risk** |
| **Bias in selection of participants into the study** | The selection of participants into the study was not based on the participant characteristics observed after the start of the intervention. Meanwhile, we are unable to know whether there was an exclusion or not in this study. | **Moderate risk** |
| **Bias in classification of interventions** | The classification and assignment of IVIg or control group were determined retrospectively. | **Moderate risk** |
| **Bias due to deviations from intended interventions** | The treatment regiments between the two groups were unbalanced. The chosen of regiment was based on the physician’s decision. | **Moderate risk** |
| **Bias due to missing data** | The primary outcomes of all included patients were reported. | **Low risk** |
| **Bias in measurement of outcomes** | The outcomes of the study were objectified (mortality, hospitalization duration and ICU duration). And the methods of outcome assessment were comparable across intervention groups. | **Low risk** |
| **Bias in selection of the reported result** | The effect estimates were not to be selected reported on the basis of the results, from multiple outcome measurements, multiple analyses of the intervention-outcome relationship, or different subgroups. | **Low risk** |
| **Overall bias** |  | **Serious risk** |

**Cao, et al. 2021**

| **Methods** | **A multicenter retrospective cohort study.** |
| --- | --- |
| **Participants** | Severe COVID-19 patients were screened in Jin-Yintan Hospital, Zhongnan Hospital of Wuhan University, and Sino-French campus of Tongji Hospital from February 7 to March 30, 2020 in Wuhan, China. |
| **Interventions** | IVIg and non-IVIg use during treatment. The total dose of IVIg was 2 g per kilogram body weight, divided over 2–5 days. |
| **Outcomes** | The primary endpoint was the 28-day mortality in the study population. Secondary clinical outcomes included time to clinical improvement after admission, defined as a reduction of two points on the seven-category ordinal scale or live discharge from the hospital, clinical status as assessed with the seven-category ordinal scale on days 7, 14, and 28, the duration of mechanical ventilation, the duration of hospitalization in survivors, the duration of positive RT-PCR results, and the time to normalizations of inflammatory factors including interleukin (IL)-6, IL-8, IL-10, tumor necrosis factor (TNF)-α, hypersensitive C-reactive protein (hsCRP), ferritin, and erythrocyte sedimentation rate (ESR). |
| **Notes** | In this study, the propensity score matching (PSM) and inverse probability of treatment weighting (IPTW) method were conducted. |

**Risk of bias table**

| **Bias** | **Support for judgement** | **Authors' judgement** |
| --- | --- | --- |
| **Bias due to confounding** | Differences in baseline and clinical characteristics were significant. Further PSM and IPTW methods were conducted to reduce the bias. | **Moderate risk** |
| **Bias in selection of participants into the study** | The selection of participants into the study was not based on the participant characteristics observed after the start of the intervention. 115 out of 907 COVID-19 patients were included in the analysis, with the exclusion of 707 mild or moderate cases and 85 cases with listed reasons. | **Low risk** |
| **Bias in classification of interventions** | The classification and assignment of IVIg or control group were determined retrospectively. | **Moderate risk** |
| **Bias due to deviations from intended interventions** | The treatment between the two groups was unbalanced. Further PSM and IPTW methods were conducted, however, the bias due to deviations from intended interventions might not be reduced. | **Moderate risk** |
| **Bias due to missing data** | The primary outcomes of all included patients were reported. In addition, for covariates with the missing values which may cause bias, the multiple imputations (MI) were used to impute the missing laboratory results. | **Low risk** |
| **Bias in measurement of outcomes** | The outcome assessors might be aware of the intervention received by study participants; however, the outcomes of the study were objectified (28-day mortality, time to clinical improvement after admission, the duration of mechanical ventilation, the duration of hospitalization in survivors, the duration of positive RT-PCR results, and the time to normalizations of inflammatory factors). And the methods of outcome assessment were comparable across intervention groups. | **Low risk** |
| **Bias in selection of the reported result** | The effect estimates were not to be selected reported on the basis of the results, from multiple outcome measurements, multiple analyses of the intervention-outcome relationship, or different subgroups. | **Low risk** |
| **Overall bias** |  | **Moderate risk** |

**Huang, et al. 2021**

| **Methods** | **A single-center retrospective cohort study.** |
| --- | --- |
| **Participants** | Non-severe patients who were admitted to Shanghai Public Health Clinical Center between January 20, 2020 and June 10, 2020. Shanghai Public Health Clinical Center is a designated hospital for the treatment of patients with COVID-19 in Shanghai, China. |
| **Interventions** | IVIg and non-IVIg use during treatment. The doses of IVIG and durations of administration were as follows: (1) 10 g/day for 3 days, 8 patients; (2) 10 g/day for 5 days, 13 patients; (3) 20 g/day for 3 days, 16 patients; (4) 20 g/day for 5 days, 8 patients. |
| **Outcomes** | The primary outcomes included the severity rate and mortality rate. Secondary outcomes included the duration of fever, virus clearance time, length of hospital stay, and use of antibiotics. |
| **Notes** | In this study, the propensity matching (PSM) method was conducted. |

**Risk of bias table**

| **Bias** | **Support for judgement** | **Authors' judgement** |
| --- | --- | --- |
| **Bias due to confounding** | Differences in baseline and clinical characteristics were significant. Patients who were older (56 vs 36 years, p < 0.001), more commonly had a comorbidity (51.1% vs 18.7%, p < 0.001), and those who had higher CRP (19.8 vs 0.5 mg/l, p < 0.001), LDH (272 vs 201 U/l, p < 0.001), and D-dimer (0.5 vs 0.3 ng/mL, p < 0.001) were more likely to be treated with IVIG. Further PSM methods were conducted to reduce the bias. | **Moderate risk** |
| **Bias in selection of participants into the study** | The selection of participants into the study was not based on the participant characteristics observed after the start of the intervention. 639 out of 664 COVID-19 patients were included in the analysis, with the exclusion of 11 severe cases and 12 cases who applied IVIg after disease progression. | **Low risk** |
| **Bias in classification of interventions** | The classification and assignment of IVIg or control group were determined retrospectively. | **Moderate risk** |
| **Bias due to deviations from intended interventions** | The treatment between the two groups was unbalanced. The corticosteroids (20% vs 7.4%, p = 0.003), thymosin α (88.9% vs 24.2%, p < 0.001), arbidol (64.4% vs 18.2%, p < 0.001), and lopinavir/ritonavir (46.7% vs 15.5%, p < 0.001) were more frequently used in the IVIG group, while hydroxychloroquine (2.2% vs 44.6%, p < 0.001) was less common in the IVIG group compared with the control group. The further PSM method was conducted, however, the bias due to deviations from intended interventions might not be reduced. | **Moderate risk** |
| **Bias due to missing data** | The detailed primary outcomes of all included patients (n= 639) were not reported. The authors reported the univariate and multivariate analysis results, which might be considered that no participants were excluded due to missing data on intervention status or due to missing data on other variables needed for the analysis. | **Moderate risk** |
| **Bias in measurement of outcomes** | The outcome assessors might be aware of the intervention received by study participants; however, the outcomes of the study were objectified (the severity rate, mortality rate, the duration of fever, virus clearance time, length of hospital stay, and use of antibiotics). And the methods of outcome assessment were comparable across intervention groups. | **Low risk** |
| **Bias in selection of the reported result** | Only the effect estimates and specific results after PSM were selected report. | **Serious risk** |
| **Overall bias** |  | **Serious risk** |

**Liu, et al. 2021**

| **Methods** | **A multicenter retrospective cohort study.** |
| --- | --- |
| **Participants** | Severe COVID-19 patients were screened in the different hospitals in China. The authors declared that this retrospective study protocol was approved by each hospital's institutional review board, which waived the requirement for informed consent, however, the detailed list of hospitals didn't show. |
| **Interventions** | IVIg and non-IVIg use during treatment. The dose of IVIg was 10 (10, 10) g/day. |
| **Outcomes** | The primary outcome was defined as 28-day all-cause mortality after propensity matching analysis. The start of the 28 days was defined as day of admission to the hospital. The secondary outcomes were defined as ARDS, DIC, myocardial injury, acute hepatic injury, shock, acute kidney injury (AKI), non-invasive mechanical ventilation, invasive mechanical ventilation, prone position ventilation, continuous renal replacement therapy and ECMO between the two groups (treated versus untreated patients). |
| **Notes** | In this study, the propensity score method was conducted to evaluate the relationship between IVIG and 28-day mortality. |

**Risk of bias table**

| **Bias** | **Support for judgement** | **Authors' judgement** |
| --- | --- | --- |
| **Bias due to confounding** | Differences in baseline and clinical characteristics were significant. Although the statistical analysis values were not shown in Table1, the differences between the two groups in Hypertension, Fever, APACHE Ⅱ score through the reported data and e-Figure 2. Further propensity score method was conducted to reduce the bias. | **Moderate risk** |
| **Bias in selection of participants into the study** | The selection of participants into the study was not based on the participant characteristics observed after the start of the intervention. 850 out of 2346 COVID-19 patients were considered severely ill, and all were included in the analysis. | **Low risk** |
| **Bias in classification of interventions** | The classification and assignment of IVIg or control group were determined retrospectively. | **Moderate risk** |
| **Bias due to deviations from intended interventions** | The treatment between the two groups was unbalanced, especially the glucocorticoid use and respiratory support. The further propensity score method was conducted, however, the bias due to deviations from intended interventions might not be reduced. | **Moderate risk** |
| **Bias due to missing data** | The primary outcomes of all included patients (IVIg, n= 421; Non-IVIg, n=429) were not reported for an unknown reason. In addition, missing data were not imputed in this study. | **Moderate risk** |
| **Bias in measurement of outcomes** | The outcome assessors might be aware of the intervention received by study participants; however, the outcomes of the study were objectified (28-day all-cause mortality, ARDS, DIC, myocardial injury, acute hepatic injury, shock, acute kidney injury (AKI), non-invasive mechanical ventilation, invasive mechanical ventilation, prone position ventilation, continuous renal replacement therapy and ECMO between the two groups). And the methods of outcome assessment were comparable across intervention groups. | **Low risk** |
| **Bias in selection of the reported result** | Only the effect estimates after PSW were selected reported on the basis of the results. | **Serious risk** |
| **Overall bias** |  | **Serious risk** |

**Hou, et al. 2021**

| **Methods** | **A single-center retrospective cohort study.** |
| --- | --- |
| **Participants** | Severe COVID-19 patients were screened in the Respiratory and Critical Care Unit of Dabie Mountain Regional Medical Center under the jurisdiction of the Shandong Medical Team. Dabie Mountain Regional Medical Center is a designated hospital in Huanggang City (Hubei Province, China) |
| **Interventions** | IVIg and non-IVIg use during treatment. However, the authors did not record the dose and course of IVIg. |
| **Outcomes** | The primary outcome was the composite end point, including death and the use of mechanical ventilation. The secondary outcome was the length of hospital stay. |
| **Notes** | The detailed dosage of IVIg was missing in this study. |

**Risk of bias table**

| **Bias** | **Support for judgement** | **Authors' judgement** |
| --- | --- | --- |
| **Bias due to confounding** | Differences in baseline and clinical characteristics were significant. Forty-seven patients (41.6%) received IVIG. Compared with patients who did not receive IVIG, fewer patients who received IVIG therapy had coronary heart disease [0 (0) vs 7 (10.6%), P = 0.021]. In addition, patients who received IVIG therapy had a higher body temperature [38.9 (38.2–39.0) vs 38.0 (37.5–38.8), P = 0.002] before hospital admission, a higher white blood cell count [7.45 (4.73–9.42) vs 5.00 (3.68–6.79), P < 0.001], a lower lymphocyte count [0.79 (0.62–1.21) vs 1.24 (0.90–1.75), P < 0.001], and a higher glutamate aminotransferase level [29.7 (18.0–47) vs 21.0 (14.2–29.2), P = 0.008], although the authors conducted the univariate logistic regression analysis was conducted to adjust the bias. | **Serious risk** |
| **Bias in selection of participants into the study** | The selection of participants into the study was not based on the participant characteristics observed after the start of the intervention. 113 out of 121 COVID-19 patients were included in the analysis, and 8 patients were excluded with reasons. | **Low risk** |
| **Bias in classification of interventions** | The selection of participants into the study was not based on the participant characteristics observed after the start of the intervention. 113 out of 121 COVID-19 patients were included in the analysis, and 8 patients were excluded with reasons. | **Moderate risk** |
| **Bias due to deviations from intended interventions** | The treatment between the two groups was unbalanced, especially the glucocorticoid use (p<0.001). More patients in the IVIG group used glucocorticoids [39 (83.0%) vs 18 (27.3%), P < 0.001], which was likely to have affected the outcome. The further multivariate analysis was conducted, however, the bias due to deviations from intended interventions might not be reduced. | **Moderate risk** |
| **Bias due to missing data** | The primary outcomes of all included patients (IVIg, n= 47; Non-IVIg, n=66) were reported. And no participants were excluded due to missing data on intervention status or due to missing data on other variables needed for the analysis. | **Low risk** |
| **Bias in measurement of outcomes** | The outcome assessors might be aware of the intervention received by study participants; however, the outcomes of the study were objectified (death, the use of mechanical ventilation, the length of hospital stay). And the methods of outcome assessment were comparable across intervention groups. | **Low risk** |
| **Bias in selection of the reported result** | The effect estimates were not to be selected reported on the basis of the results, from multiple outcome measurements, multiple analyses of the intervention-outcome relationship, or different subgroups. | **Low risk** |
| **Overall bias** |  | **Serious risk** |

**Ali, et al. 2021**

| **Methods** | **A single-center retrospective cohort study.** |
| --- | --- |
| **Participants** | Critically ill patients who were admitted to ICUs at Hazm Mebaireek General Hospital, Qatar, between March 7, 2020 and September 9, 2020. |
| **Interventions** | IVIg and non-IVIg use during treatment. The IVIG treatment group received a minimum one dose of 0.4 g/kg of IVIG. Further doses of IVIG were given on consecutive days, to a maximum of 5 doses, based on the treating physician's decision. |
| **Outcomes** | The primary outcome was all-cause ICU mortality. Secondary outcomes studied were ventilator-free days and ICU-free days at day-28, and incidence of acute kidney injury (AKI). |
| **Notes** | In this study, propensity score matching (PSM) was used to adjust for confounders, and the primary outcome was compared using competing-risks survival analysis. |

**Risk of bias table**

| **Bias** | **Support for judgement** | **Authors' judgement** |
| --- | --- | --- |
| **Bias due to confounding** | Differences in baseline and clinical characteristics were significant. The patients in the IVIG group were older, had a higher prevalence of hypertension, dyslipidemia, and hemodialysis, had lower PaO2/FiO2 ratio, lesser vasopressor use, lower SOFA score, raised alanine transaminase (ALT). Further PSM analysis was conducted to reduce the bias. | **Moderate risk** |
| **Bias in selection of participants into the study** | The selection of participants into the study was not based on the participant characteristics observed after the start of the intervention. However, only 590 out of 787 COVID-19 patients who needed invasive ventilation in ICU were included in the analysis, and the start of follow-up and start of intervention might not coincide for most participants. | **Moderate risk** |
| **Bias in classification of interventions** | The classification and assignment of IVIg or control group were determined retrospectively. | **Moderate risk** |
| **Bias due to deviations from intended interventions** | The treatment between two groups was unbalanced, especially the glucocorticoid use (p<0.001) which was likely to have affected the outcome. The further PSM method was conducted, however, the bias due to deviations from intended interventions might not be reduced. | **Moderate risk** |
| **Bias due to missing data** | The primary outcomes of all included patients (n=590 with all-cause ICU mortality for 27.1%) were reported. And no participants were excluded due to missing data on intervention status or due to missing data on other variables needed for the analysis. | **Low risk** |
| **Bias in measurement of outcomes** | The outcome assessors might be aware of the intervention received by study participants; however, the outcomes of the study were objectified (The primary outcome was all-cause ICU mortality. Secondary outcomes studied were ventilator-free days and ICU-free days at day-28, and incidence of AKI). And the methods of outcome assessment were comparable across intervention groups. | **Low risk** |
| **Bias in selection of the reported result** | Only the effect estimates among PSM and PS-adjusted cohort were selected reported on the basis of the results. | **Serious risk** |
| **Overall bias** |  | **Serious risk** |

**Kilic, et al. 2021**

| **Methods** | **A single-center retrospective cohort study.** |
| --- | --- |
| **Participants** | Severe COVID-19 patients at Edirne Sultan 1. Murat State Hospital who were treated with or without high dose IVIG in ICU were enrolled |
| **Interventions** | IVIg and non-IVIg use during treatment. The dosage and duration of IVIg therapy was 0.3–0.5 g per kg weight daily for five days. |
| **Outcomes** | 28 days of ICU mortality. |
| **Notes** | This study did not report the adjusted RR of IVIg therapy on mortality, only the unadjusted OR with 95% CI was reported. |

**Risk of bias table**

| **Bias** | **Support for judgement** | **Authors' judgement** |
| --- | --- | --- |
| **Bias due to confounding** | Differences in baseline clinical characteristics were significant. The baseline levels of CRP and NLR were much higher in the IVIg group and the number of LYM was also significant lower (0.2 (0.1-0.4) vs. 1.1 (0.6-1.2), p = 0.001). | **Serious risk** |
| **Bias in selection of participants into the study** | The selection of participants into the study was not based on the participant characteristics observed after the start of the intervention. Meanwhile, we are unable to know whether there was an exclusion or not in this study. | **Moderate risk** |
| **Bias in classification of interventions** | The classification and assignment of IVIg or control group were determined retrospectively. | **Moderate risk** |
| **Bias due to deviations from intended interventions** | The treatment difference between the two groups wasn't reported. | **Moderate risk** |
| **Bias due to missing data** | The study reported the outcomes of all included patients (IVIg, n=10; Non-IVIg, n=16). | **Low risk** |
| **Bias in measurement of outcomes** | The outcome assessors might be aware of the intervention received by study participants; however, the outcomes of the study were objectified (mortality, in‐hospital days and in-ICU days). The methods of outcome assessment were comparable across intervention groups. | **Low risk** |
| **Bias in selection of the reported result** | The effect estimates were not to be selected reported on the basis of the results, from multiple outcome measurements, multiple analyses of the intervention-outcome relationship, or different subgroups. | **Low risk** |
| **Overall bias** |  | **Serious risk** |

**Chen, et al. 2022**

| **Methods** | **A multicenter retrospective cohort study.** |
| --- | --- |
| **Participants** | Critically ill patients who were admitted to intensive care units (ICUs) of the participating hospitals between January 1 and February 29, 2020 in 19 hospitals in Wuhan (Hubei Province), Huangshi (Hubei Province), Shenzhen (Guangdong Province), and Jiangsu, China. |
| **Interventions** | IVIg and non-IVIg use during treatment. However, the authors did not consider the dose and course of IVIG, although they declared most patients were administered with a conventional dose of 0.5 g/kg/day. |
| **Outcomes** | The primary outcome was 28-day mortality. |
| **Notes** | In this study, the propensity score matching (PSM) and the inverse probability of treatment weighting (IPTW) analysis were conducted. However, the detailed dosage of IVIg was missing in this study. |

**Risk of bias table**

| **Bias** | **Support for judgement** | **Authors' judgement** |
| --- | --- | --- |
| **Bias due to confounding** | Differences in baseline and clinical characteristics were significant. The days from illness onset to ICU admission were longer in the non-IVIG group compared with the IVIG group (13 (IQR 9, 20) vs. 12 (IQR 8, 17), p = 0.009). More patients in the non-IVIG group than in the IVIG group required vasopressin on ICU admission (47% vs. 31%, p = 0.027). Further PSM and IPTW were conducted to reduce bias | **Moderate risk** |
| **Bias in selection of participants into the study** | The selection of participants into the study was not based on the participant characteristics observed after the start of the intervention. Besides, 7 patients were excluded for missing records of IVIg use data among 761 ICU cases. The rest 754 patients with eligibility were all included for analysis. | **Low risk** |
| **Bias in classification of interventions** | The classification and assignment of IVIg or control group were determined retrospectively. | **Moderate risk** |
| **Bias due to deviations from intended interventions** | The treatment between the two groups was unbalanced, especially the glucocorticoid use (p<0.001) which was likely to have affected the outcome. In addition, the IVIg dosage was missing in this study, which would introduce the extra deviations from intended interventions bias. Further PSM and IPTW methods were conducted, however, the bias due to deviations from intended interventions might not be reduced. | **Moderate risk** |
| **Bias due to missing data** | The primary outcomes of all included patients (IVIg, n=392; Non-IVIg, n=362) were reported. And no participants were excluded due to missing data on intervention status or due to missing data on other variables needed for the analysis. | **Low risk** |
| **Bias in measurement of outcomes** | The outcome assessors might be aware of the intervention received by study participants; however, the outcomes of the study were objectified (28-day mortality). And the methods of outcome assessment were comparable across intervention groups. | **Low risk** |
| **Bias in selection of the reported result** | The effect estimates were not to be selected reported on the basis of the results, from multiple outcome measurements, multiple analyses of the intervention-outcome relationship, or different subgroups. | **Low risk** |
| **Overall bias** |  | **Moderate risk** |

**Salehi, et al. 2022**

| **Methods** | **A multicenter retrospective cohort study.** |
| --- | --- |
| **Participants** | Critically ill patients who were admitted to ICU between February 2020 and December 2020 in Imam Khomeini hospital complex, Rasul-e Akram, and Ziaeian hospitals in Tehran, Iran. |
| **Interventions** | IVIg and non-IVIg use during treatment. The IVIg group was divided into three groups of the low, medium, and high doses (0.25, 0.5, and 1 gr/kg) during 3–5 consecutive days in addition to standard care. The standard treatment group regimen was oral hydroxychloroquine (HCQ) 400 mg daily for 5 days plus atazanavir/ritonavir (300/100) daily for 10 days. |
| **Outcomes** | Primary outcomes including mortality rate, duration of hospitalization, ICU length of stay, and duration of mechanical ventilation were compared in the two groups |
| **Notes** | The administration of IVIg dosage (low, moderate, high dose) and IVIg time was different, the subgroup analysis was conducted. |

**Risk of bias table**

| **Bias** | **Support for judgement** | **Authors' judgement** |
| --- | --- | --- |
| **Bias due to confounding** | There were no significant differences between the two groups in demographic data, vital signs on admission, clinical features, laboratory tests, and even risk factors. The study can be considered to be at low risk of bias due to confounding and no further signaling questions need to be considered | **Low risk** |
| **Bias in selection of participants into the study** | The selection of participants into the study was not based on the participant characteristics observed after the start of the intervention. Besides, 19 patients were excluded for unavailable data among 202 ICU cases. The rest 183 patients with eligibility were all included for analysis. | **Low risk** |
| **Bias in classification of interventions** | The classification and assignment of IVIg or control group were determined retrospectively. | **Moderate risk** |
| **Bias due to deviations from intended interventions** | The treatment intervention between the IVIg group and the control group was the application of IVIg. | **Low risk** |
| **Bias due to missing data** | The primary and secondary outcomes of all included patients (IVIg, n=74; Non-IVIg, n=109) were reported. And no participants were excluded due to missing data on intervention status or due to missing data on other variables needed for the analysis. | **Low risk** |
| **Bias in measurement of outcomes** | The outcome assessors might be aware of the intervention received by study participants; however, the outcomes of the study were objectified (mortality rate, duration of hospitalization, ICU length of stay, and duration of mechanical ventilation). And the methods of outcome assessment were comparable across intervention groups. | **Low risk** |
| **Bias in selection of the reported result** | The effect estimates were not to be selected reported on the basis of the results, from multiple outcome measurements, multiple analyses of the intervention-outcome relationship, or different subgroups. | **Low risk** |
| **Overall bias** |  | **Moderate risk** |

**Aggarwal, et al. 2022**

| **Methods** | **A single-center retrospective cohort study.** |
| --- | --- |
| **Participants** | Severe and critical COVID-19 patients were admitted to the ICU from May 2020 to December 2020 in Max Smart Super Specialty Hospital, New Delhi, India. |
| **Interventions** | IVIg and non-IVIg use during treatment. The dose of IVIG was 0.5 g/kg body weight/day as a continuous infusion for 3 days. The actual body weight of the patient was taken for calculating the dose. All patients received a uniform dose and uniform duration of IVIG treatment. |
| **Outcomes** | The primary outcome of the study was the percentage of patients requiring mechanical ventilation. Secondary outcome measures included in-hospital mortality, 28-day mortality, ICU-length of stay (ICU-LOS), days to discontinuation of supplemental oxygen, and days to COVID RT-PCR negativity. |
| **Notes** | The administration time of IVIg was different (≤7 days or >7 days from ICU admission), the subgroup analysis and multivariate analysis were conducted to adjust. |

**Risk of bias table**

| **Bias** | **Support for judgement** | **Authors' judgement** |
| --- | --- | --- |
| **Bias due to confounding** | The characteristics of included IVIg group and control group were imbalanced at baseline. The profile of comorbidities in the two groups was similar except for Diabetes (57.3% vs 47.9%, p = 0.03) and Hypertension (64.7% vs 47.1%, p < 0.0001) which were significantly more in those in the IVIG group. Even the subgroup analysis and multivariate analysis were conducted to adjust the effect, there might be bias. | **Serious risk** |
| **Bias in selection of participants into the study** | The selection of participants into the study was not based on the participant characteristics observed after the start of the intervention. However, this study did exclude patients that received IVIg that less 3 days course (n=29) and patients that received plasma treatment after taking IVIg (n=42), whether these would introduce the selection bias remained unclear. | **Moderate risk** |
| **Bias in classification of interventions** | The classification and assignment of IVIg or control group were determined retrospectively. | **Moderate risk** |
| **Bias due to deviations from intended interventions** | The treatment difference between the two groups wasn't reported, although the authors mentioned that patients treated with high-dose steroids (>1 mg/kg methylprednisolone) were excluded. | **Moderate risk** |
| **Bias due to missing data** | The primary and secondary outcomes of all included patients (IVIg, n= 255; Non-IVIg, n=280) were reported. And no participants were excluded due to missing data on intervention status or due to missing data on other variables needed for the analysis. | **Low risk** |
| **Bias in measurement of outcomes** | The outcome assessors might be aware of the intervention received by study participants; however, the outcomes of the study were objectified (the percentage of patients requiring mechanical ventilation. Secondary outcome measures included in-hospital mortality, 28-day mortality, ICU-length of stay (ICU-LOS), days to discontinuation of supplemental oxygen, and days to COVID RT-PCR negativity). And the methods of outcome assessment were comparable across intervention groups. | **Low risk** |
| **Bias in selection of the reported result** | The effect estimates were not to be selected reported on the basis of the results, from multiple outcome measurements, multiple analyses of the intervention-outcome relationship, or different subgroups. | **Low risk** |
| **Overall bias** |  | **Serious risk** |
